# Echinomycin inhibits adipogenesis in 3T3-L1 cells in a HIF-independent manner

**DOI:** 10.1038/s41598-017-06761-4

**Published:** 2017-07-26

**Authors:** Junna Yamaguchi, Tetsuhiro Tanaka, Hisako Saito, Seitaro Nomura, Hiroyuki Aburatani, Hironori Waki, Takashi Kadowaki, Masaomi Nangaku

**Affiliations:** 1 Division of Nephrology and Endocrinology, The University of Tokyo Graduate School of Medicine, Tokyo, Japan; 2 Genome Science laboratory, Research Center for Advanced Science and Technology, The University of Tokyo, Tokyo, Japan; 3 Department of Diabetes and Metabolic Diseases, Department of Molecular Science on Diabetes, Graduate School of Medicine, The University of Tokyo, Tokyo, Japan; 4 Department of Diabetes and Metabolic Diseases, Graduate School of Medicine, The University of Tokyo, Tokyo, Japan

## Abstract

Obesity is a risk factor for many diseases including diabetes, cancer, cardiovascular disease, and chronic kidney disease. Obesity is characterized by the expansion of white adipose tissue (WAT). Hypertrophy and hyperplasia of adipocytes cause tissue hypoxia followed by inflammation and fibrosis. Its trigger, preadipocyte differentiation into mature adipocytes, is finely regulated by transcription factors, signal molecules, and cofactors. We found that echinomycin, a potent HIF-1 inhibitor, completely inhibited adipogenesis in 3T3-L1 WAT preadipocytes by affecting the early phase of mitotic clonal expansion. The dose required to exert the effect was surprisingly low and the time was short. Interestingly, its inhibitory effect was independent of HIF-1 pathways. Time-course DNA microarray analysis of drug-treated and untreated preadipocytes extracted a major transcription factor, CCAAT/enhancer-protein β, as a key target of echinomycin. Echinomycin also inhibited adipogenesis and body weight gain in high fat diet mice. These findings highlight a novel role of echinomycin in suppressing adipocyte differentiation and offer a new therapeutic strategy against obesity and diabetes.

## Introduction

Obesity is a global health burden and serves as a significant risk factor for many diseases such as diabetes, hypertension, cancer, cardiovascular disease, and chronic kidney disease^1, 2^. Expansion of white adipose tissue (WAT), which is caused by the proliferation and differentiation of white adipocytes, is responsible for obesity development. Elucidating the underlying mechanism and abrogating it have been of great interest in this field.

Obesity exacerbates tissue hypoxia due to increased adipocyte oxygen consumption and insufficient angiogenesis^3, 4^. Hypoxia inducible factor (HIF)-1, the master regulator of cellular adaptation to hypoxia consisting of α and β subunits, is induced early in the course of diet-induced obese WAT^4^ and contributes to glucose intolerance and insulin resistance^4, 5^. Previous reports on genetic deletion of HIF-1 have both positive and negative effects on adipogenesis of WAT^4, 5^.

Echinomycin is a cyclic peptide belonging to a family of quinoxaline antibiotics isolated from *Streptomyces echinatus* ^6^. It reversibly intercalates into double-stranded DNA sequences such as 5′-CGTACG, 5′-[d(ACGTACGT)2] or 5′-[d(TCGATCGA)2]. The target sequence includes a hypoxia responsive element (HRE) sequence, making echinomycin a potent HIF-1 inhibitor^7, 8^. Antitumour activity was confirmed in several preclinical studies, which led to a phase II clinical trial of echinomycin in metastatic soft tissue sarcoma patients^9^.

We decided to investigate the effect and mechanism of echinomycin as an HIF-1 inhibitor of WAT adipogenesis in 3T3-L1 cells, a preadipocyte cell line that is widely used as a model of adipocyte differentiation with a well characterized adipogenic cascade^1, 2^, as well as in high fat diet (HFD) mice that is commonly used as an obesity model.

## Results

### Echinomycin Inhibits Adipogenesis in 3T3-L1 Cells

**Figure 1:**
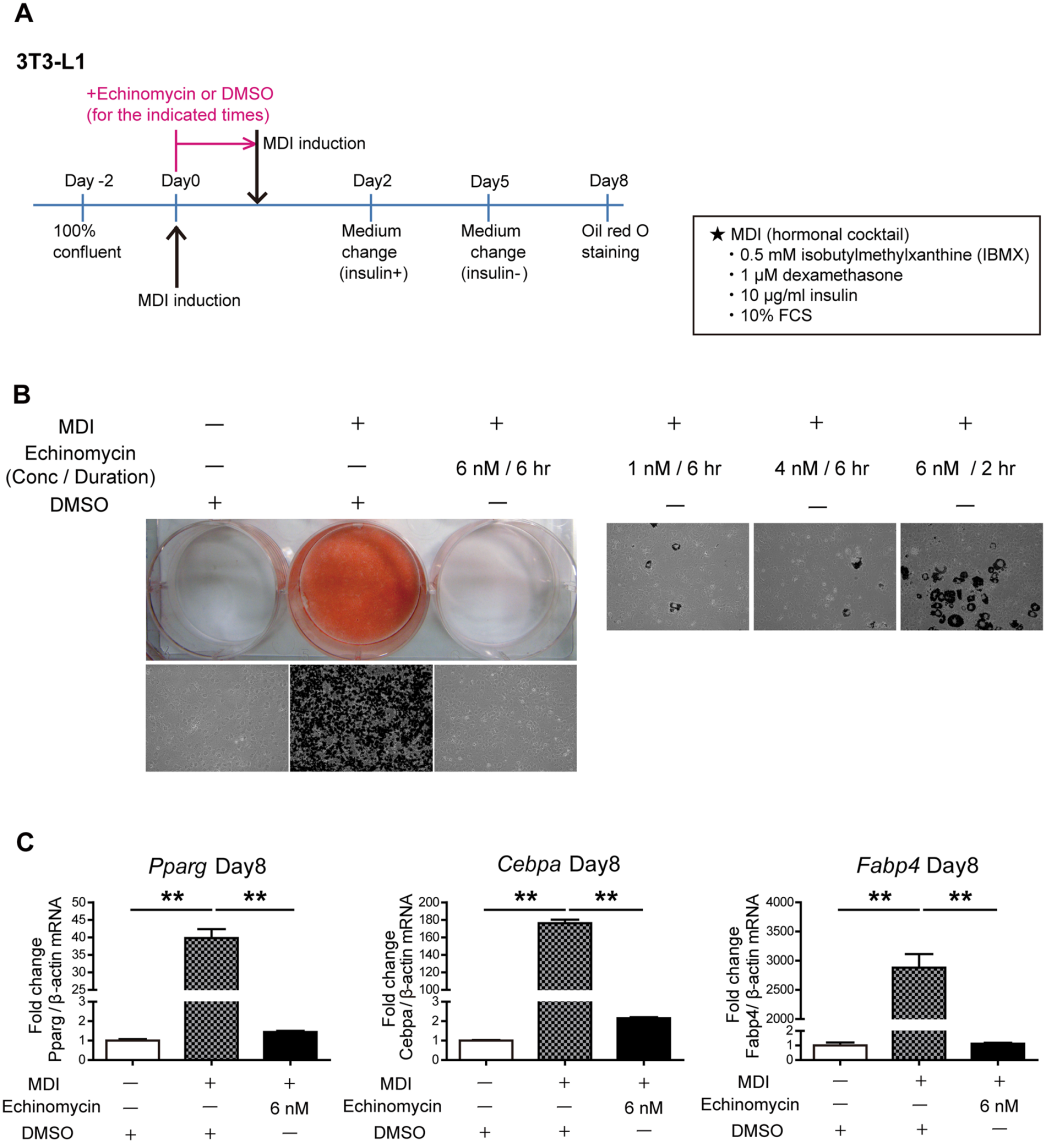
Echinomycin inhibits adipogenesis in 3T3-L1 cells in a time- and dose-dependent manner. (**A**) Protocol for preadipocyte differentiation and echinomycin treatment: two days after 3T3-L1 cells reached full confluence, they were treated with medium containing a differentiation cocktail containing MDI [10% FCS, 3-isobutyl-1-methylxanthine (0.5 mM), dexamethasone (1 μM), and insulin (10 μg/ml)] for 2 days, and then, for an additional 3 days, the cells were maintained in DMEM containing 10% FCS and insulin (10 μg/ml). Echinomycin or DMSO was added to medium with MDI, and after the times (hr) indicated in each experiment, cells were gently washed to remove echinomycin or DMSO, and the medium was exchanged with fresh medium containing MDI. (**B**) Oil red O staining 8 days after induction. (*Left upper panel*) Macroscopic shots of oil red O staining are shown. (*Left lower panel*) 10x white-black converted shots are presented. The red areas in the colour pictures, which correspond to triglyceride lipid droplets, were converted to black areas for easier comprehension. (*Right panel*) Lower concentrations or shorter durations of echinomycin treatment of 3T3-L1 cells result in insufficient adipogenesis inhibition, demonstrated by the same method as *left lower panel* (20x white-black converted shots of oil red O staining at day 8). (**C**) qRT-PCR analysis of mature adipocyte gene expression in 3T3-L1 cells at day 8. Mature adipogenic genes such as *Pparg*, *Cebpa*, and *Fabp4* were induced by MDI and suppressed by echinomycin treatment. The data are expressed as the mean ± SEM from three independent experiments. **P < 0.01, as determined by one-way ANOVA with Tukey’s post-test.

The conventional hormone cocktails containing MDI lead 3T3-L1 preadipocytes to robustly differentiate into mature adipocytes as shown by a substantial amount of lipid accumulation in mature adipocytes in oil red O staining (Fig. 1A and B). To investigate the effect of echinomycin on adipocyte differentiation, 3T3-L1 preadipocytes were treated with various concentrations and/or durations of echinomycin simultaneously with MDI (Fig. 1B). Echinomycin inhibited adipogenesis in a time- and dose-dependent manner. Even under re-stimulation with MDI 6 hr after MDI plus echinomycin treatment, cells failed to differentiate into mature adipocytes (Fig. 1B *left upper panel*). Treatment with 6 nM for 6 hr was sufficient to fully inhibit adipogenesis, and we used this condition in the following studies unless otherwise indicated. This treatment concentration and time is within the range of previously reported usage of echinomycin in cancer cells (e.g., U-251 and HeLa cells)^7, 8, 10^. Accordingly, the altered expression levels of mature adipogenic marker genes such as fatty acid binding protein 4 (*Fabp4*), peroxisome proliferator-activated receptor γ (*Pparg*), and CCAAT/enhancer-binding protein α (*Cebpa*) were blunted by echinomycin treatment (Fig. 1C). On the other hand, the addition of echinomycin from 24 hr after induction was insufficient to abolish adipocyte differentiation (data not shown), which suggested that echinomycin exerts its effect on the early stage of adipogenesis.

### Echinomycin Inhibits Adipogenesis Independently of HIF Activity

**Figure 2:**
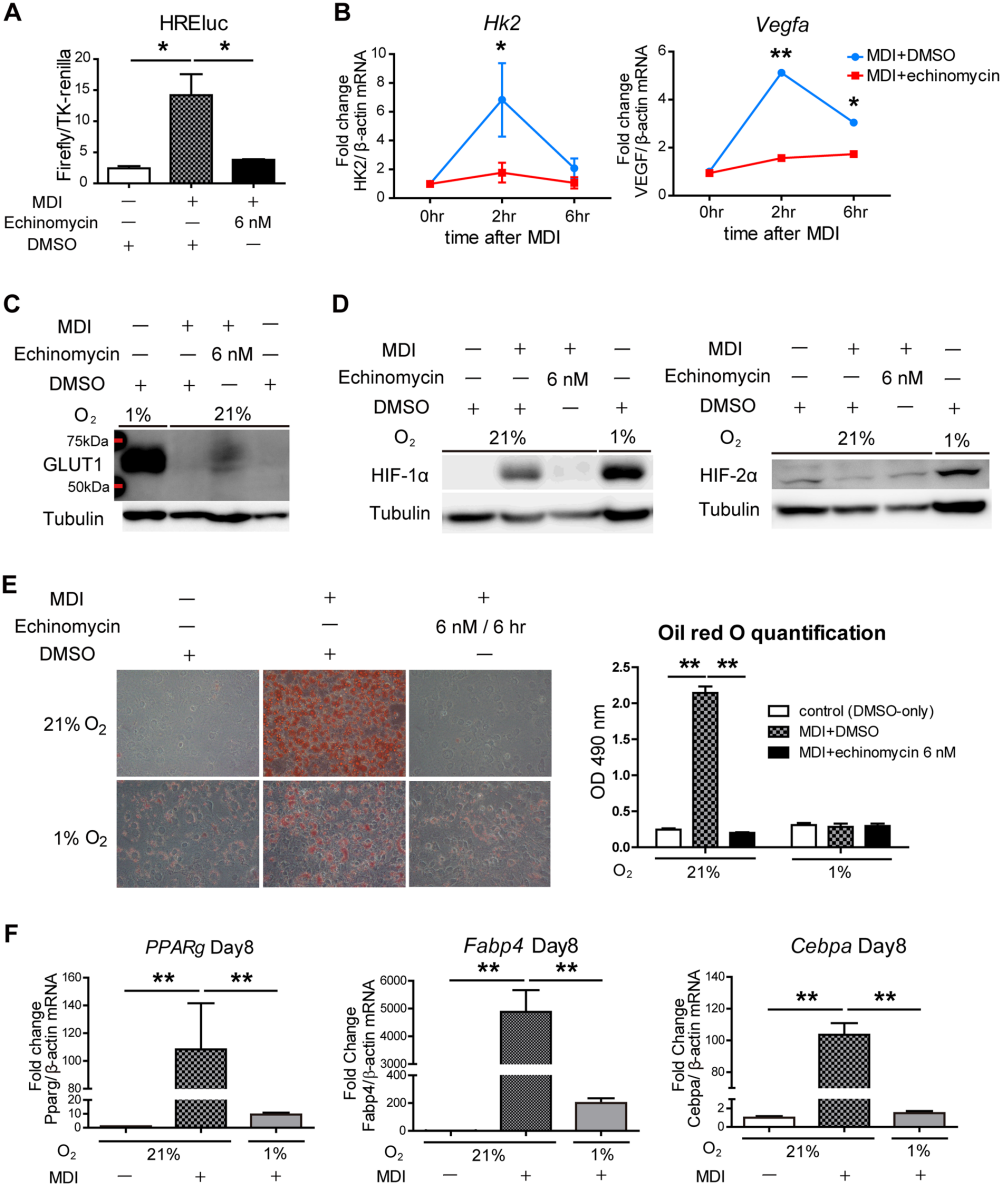
Echinomycin inhibits adipogenesis independently of the HIF-1 pathway. (**A**) The ratio of HRE luciferase (HREluc) reporter activity (firefly/TK-renilla) was measured at 8 hr after induction with or without MDI and/or echinomycin treatment. The data are the mean ± SEM of three independent experiments. *P < 0.05 vs. control (only-DMSO treatment), as determined by a one-way ANOVA with Tukey’s post-test. (**B**) qRT-PCR for representative HIF-1 target genes such as *Hk2* and *Vegfa* at 6 hr after MDI treatment. The data are the mean ± SEM of three independent experiments. *P < 0.05, **P < 0.01, as determined by a two-way ANOVA with Bonferroni’s post-test. (**C**) Immunoblot analysis of a HIF-1 target gene, GLUT1, in 3T3-L1 cells at 6 hr after treatment with MDI + DMSO or MDI + echinomycin (6 nM). 3T3-L1 cells under hypoxia (1% O_2_) as the positive control. Full-length blots are presented in Supplementary Figure. (**D**) Immunoblot analysis of HIF-1α and HIF-2α in 3T3-L1 cells at 2 hr after treatment with MDI + DMSO or MDI + echinomycin (6 nM). 3T3-L1 cells under hypoxia (1% O_2_) as the positive control. Full-length blots are presented in Supplementary Figure. (**D**) 20x images of oil red O staining at 8 days after adipogenic treatment ± echinomycin (6 nM for 6 hr) under normoxia (21% O_2_) or hypoxia (1% O_2_). Quantification of the Oil red O staining was performed by eluting the dye with isopropanol and measure the OD 490 nm absorbance. The quantification data are the mean ± SEM of three independent experiments. *P < 0.05, **P < 0.01, as determined by a two-way ANOVA with Bonferroni’s post-test. (**F**) qRT-PCR analysis of mature adipocyte gene expression in 3T3-L1 cells at day 8 under hypoxia. Mature adipogenic genes such as *Pparg*, *Cebpa*, and *Fabp4* induced by MDI were suppressed by hypoxic condition (1% O_2_). The data are expressed as the mean ± SEM from three independent experiments. **P < 0.01, as determined by one-way ANOVA with Tukey’s post-test.

Echinomycin intercalates DNA and binds to sequences including HRE sequences, that is, GCGTG or ACGTG, inhibiting HIF transcriptional activity. In 3T3-L1 cells, 6 nM echinomycin treatment significantly reduced the increased HREluc reporter activity from MDI treatment (Fig. 2A), as well as the expression levels of representative HIF-1 target genes such as hexokinase 2 (*Hk2*), vascular endothelial growth factor A (*Vegfa*) (Fig. 2B), and glucose transporter 1 (GLUT1) (Fig. 2C). Although HIF-1α was induced at 2 hr after MDI treatment, which was probably a PI3K/AKT-mediated response to insulin, this induction was very modest compared with that by hypoxic stimuli (Fig. 2D). Although echinomycin is a DNA intercalater, it also affects the expression level of HIF-1α protein depending on the cell line^11^; in 3T3-L1 cells, echinomycin reduced HIF-1α protein expression (Fig. 2D *left panel*). HIF-2α was not induced at this early time point (Fig. 2D *right panel*), which is in accordance with previous reports that HIF-2α is induced in much later phase of adipogenesis^12, 13^. These results demonstrate that the conventional effect of echinomycin as a HIF-1 inhibitor both in function and quantity also applies to 3T3-L1 cells.

On the other hand, hypoxia (1% O_2_) inhibited adipocyte differentiation, which was demonstrated by Oil red O staining and its quantification (Fig. 2E). qRT-PCR for the mature adipocyte marker genes (e.g., *Pparg*, *Fabp4*, and *Cebpa*) at day 8 after MDI exposure also remained low under hypoxic condition (Fig. 2F). Previous studies have also reported that 3T3-L1 cells with knockdown of HIF-1α undergo adipogenic differentiation even under hypoxia, which implies that HIF-1 functions as an inhibitory signal in the cascade^12, 14^. Thus, if an effect of echinomycin on adipogenesis is mediated by the HIF pathway, echinomycin should have stimulated adipogenesis. Our results suggest that echinomycin inhibits adipogenesis independently of HIF pathways.

### Transcriptome Analysis of Early Adipogenesis with or without Echinomycin

**Figure 3:**
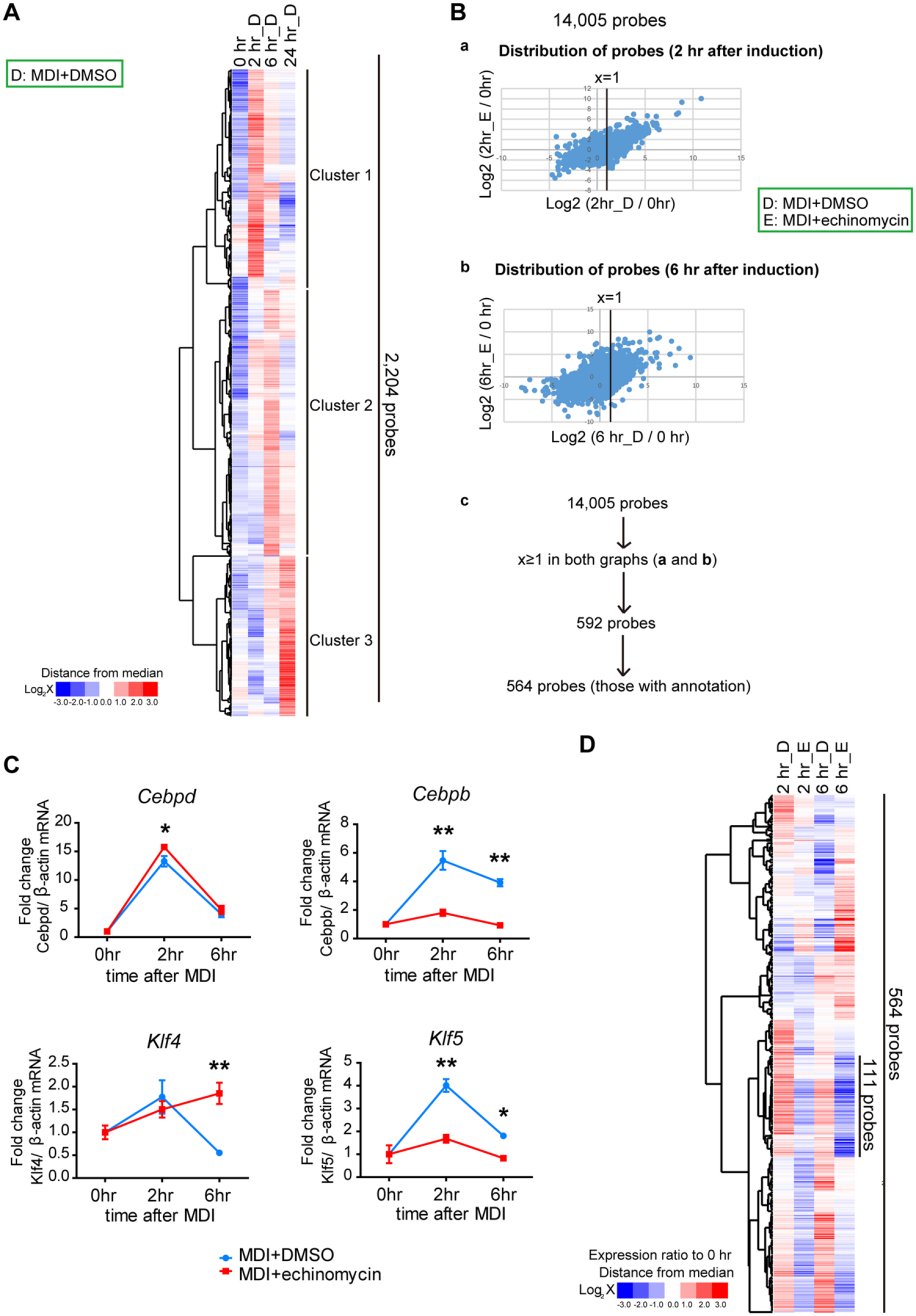
Time-course DNA microarray analysis of 3T3-L1 cells treated with MDI plus DMSO or MDI plus echinomycin. (**A**) Clustering analysis performed among the MDI + DMSO treated groups using 2,204 selected probes that had a fold change ≥ 2.0 in expression compared with the control (0 hr) (See the details in Methods). Genes were clustered into 3 groups according to their timing of response to MDI treatment. (**B**) (a,b) Scatter plot of all the 14,005 probes (signal intensity ≥ 100 at any one point) at 2 hr and 6 hr. A log2-fold change from the control (time 0 hr) of the MDI + echinomycin group is plotted against a log2-fold change from the control (time 0 hr) of the MDI + DMSO group. (c) The scheme of probe selection for further clustering. Genes that had a fold change ≥ 2.0 (x ≥ 1) at both 2 hr and 6 hr relative to time 0 preadipocytes were further analysed using hierarchical clustering. (**C**) Representative quantitative RT-PCR analysis of *Cebpb*, *Cebpd*, *Klf4*, and *Klf5* were performed to validate the microarray analysis. The data are the mean ± SEM of three independent experiments. *P < 0.05, **P < 0.01, as determined by a two-way ANOVA with Bonferroni’s post-test. (**D**) A heatmap of the expression ratio (compared with 0 hr time point) is shown, and 111 probes are extracted as a cluster containing the genes induced by MDI and suppressed by echinomycin at both 2 hr and 6 hr.

To investigate the molecular mechanism that underlies the effect of echinomycin on adipogenesis, gene expression profiling of 3T3-L1 cells during adipogenesis was performed with DNA microarrays at times 0, 2, 6, and 24 hr following treatment either with MDI plus DMSO or MDI plus echinomycin (6 nM for 6 hr). First, the 2,204 gene set probes that increased more than 2-fold compared with 0 hr among the time-course DMSO-treated control groups were selected and clustered into three groups (Fig. 3A and Table S1). Different subsets of genes responded at different time points to MDI stimulation, consistent with previous reports^15^; cluster 1 with genes responded as early as 2 hr after MDI treatment [e.g., CCAAT/enhancer-binding protein (*Cebp*) *b*, *Cebpd*, Kruppel like factor (*Klf*) *4*, *Klf5*, and *Klf9*]; cluster 2 with genes such as fos-like antigen 1 (*Fosl1*) and integrin subunit alpha 5 (*Itga5*); and cluster 3 with genes such as a disintegrin, metallopeptidase domain 8 (*Adam8*) and hepatocyte growth factor (*Hgf*).

To focus on the gene expression profiles and their changes at the very early phase of adipogenesis, we compared gene expression profiles of cells treated with MDI plus DMSO and MDI plus echinomycin at 2 hr and 6 hr after induction. The 14,005 probes were distributed in all of the four quadrants at either 2 hr or 6 hr, which signified that some of the genes are even upregulated under echinomycin treatment, ruling out the possibility that echinomycin globally suppresses gene transcription (Fig. 3B). The microarray analysis was validated by qRT-PCR for representative genes in adipogenesis (e.g., *Cebpb*, *Cebpd*, *Klf4*, and *Klf5*) (Fig. 3C). The probes induced in both the 2 hr- and 6 hr- MDI plus DMSO treated groups more than 2-fold compared with 0 hr were extracted (Fig. 3B-c). The resulting 564 probes with annotation (out of 592 probes) were further clustered by centred average linkage (Fig. 3D and Table S2). Because a 2 hr treatment with echinomycin was insufficient to inhibit adipogenesis (Fig. 1B), expression of the target genes of echinomycin was expected to be suppressed at both 2 hr and 6 hr. The cluster that met this criterion included 111 probes (Fig. 3D and Table S2).

### C/EBPβ as a Key Target of Echinomycin

**Figure 4:**
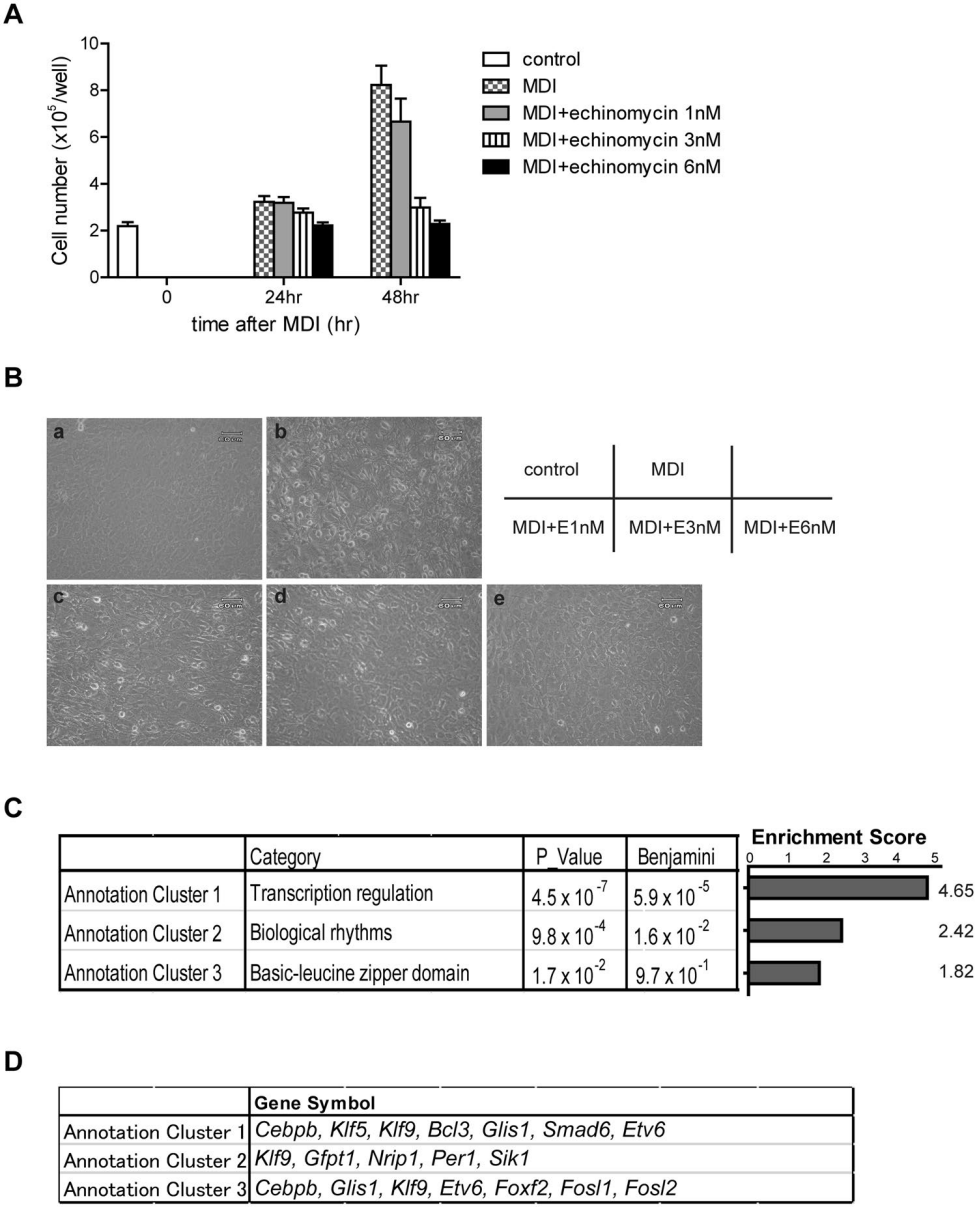
Echinomycin inhibits transcription and cell proliferation in 3T3-L1 cells. (**A**) Time-course cell counts were performed using trypan blue staining at 0, 24, and 48 hr after treatment with MDI + DMSO or MDI + echinomycin. Echinomycin inhibited cell proliferation in a dose-dependent manner. The data are the mean ± SEM of three independent experiments. (**B**) Microscopic view (20x) of 3T3-L1 cells at 24 hr after MDI (+DMSO or echinomycin) treatment. (**C**) Significantly enriched functional clusters (defined as Enrichment Score > 1.3, equivalent to non-log scale 0.05.) as determined by gene ontology analysis of the 111 probes by DAVID (listed in Fig. 3D and Table S1). (**D**) The representative gene symbols of each group in Fig. 4C are shown.

During early differentiation (24–48 hr following MDI treatment), 3T3-L1 cells are known to re-enter the cell cycle and undergo two to three cycles of clonal expansion before terminal differentiation^1, 16, 17^. The fact that re-stimulation with MDI 6 hr after echinomycin treatment failed to induce adipocyte differentiation supports the hypothesis that echinomycin inhibited this critical phase of clonal expansion. We observed that cell proliferation was inhibited in an echinomycin-dose-dependent manner in this period, supporting our hypothesis (Fig. 4A and B). Gene ontology analysis of the above 111 probes by DAVID also showed significant enrichment (enrichment score 4.65, *P* < 4.5 × 10^−7^) in transcriptional regulation (Fig. 4D). Representative genes included *Cebpb*, *Klf5*, *Klf9*, B-cell lymphoma 3-encoded protein (*Bcl3*), Glis Family Zinc Finger 1 (*Glis1*), *Smad6*, and *Etv6*. These results suggest that the key transcription factors critical in the adipogenic cascade are targets of echinomycin.

Three members of the C/EBP family, C/EBPβ, C/EBPδ and C/EBPα, play crucial roles in adipocyte differentiation^18, 19^. In the very early phase of adipogenesis, C/EBPβ and C/EBPδ are induced, and these in turn activate PPARγ and C/EBPα, which play central roles in the rest of the regulatory cascade in adipocyte differentiation^20–22^. Mice lacking C/EBPβ display reduced depots of WAT, a phenotype exacerbated in C/EBPβ−/−/C/EBPδ−/− mice^23^. Whereas *Cebpb* was extracted as one of the top candidate target genes of echinomycin, the expression level of *Cebpd* did not change following echinomycin treatment (Fig. 3C and Table S2). Other adipogenic cascade regulators such as KLF5 and KLF9, which are listed as candidate genes in Fig. 4D, are known to be downstream of C/EBPβ and thus are not expected to be a direct target of echinomycin. In addition, genes upstream of *Cebpb* in the cascade, such as *Creb*, *Klf4*, and *Krox20*, were not affected by echinomycin treatment either (Figs 3C and 4C, and Table S2)^24^.

**Figure 5:**
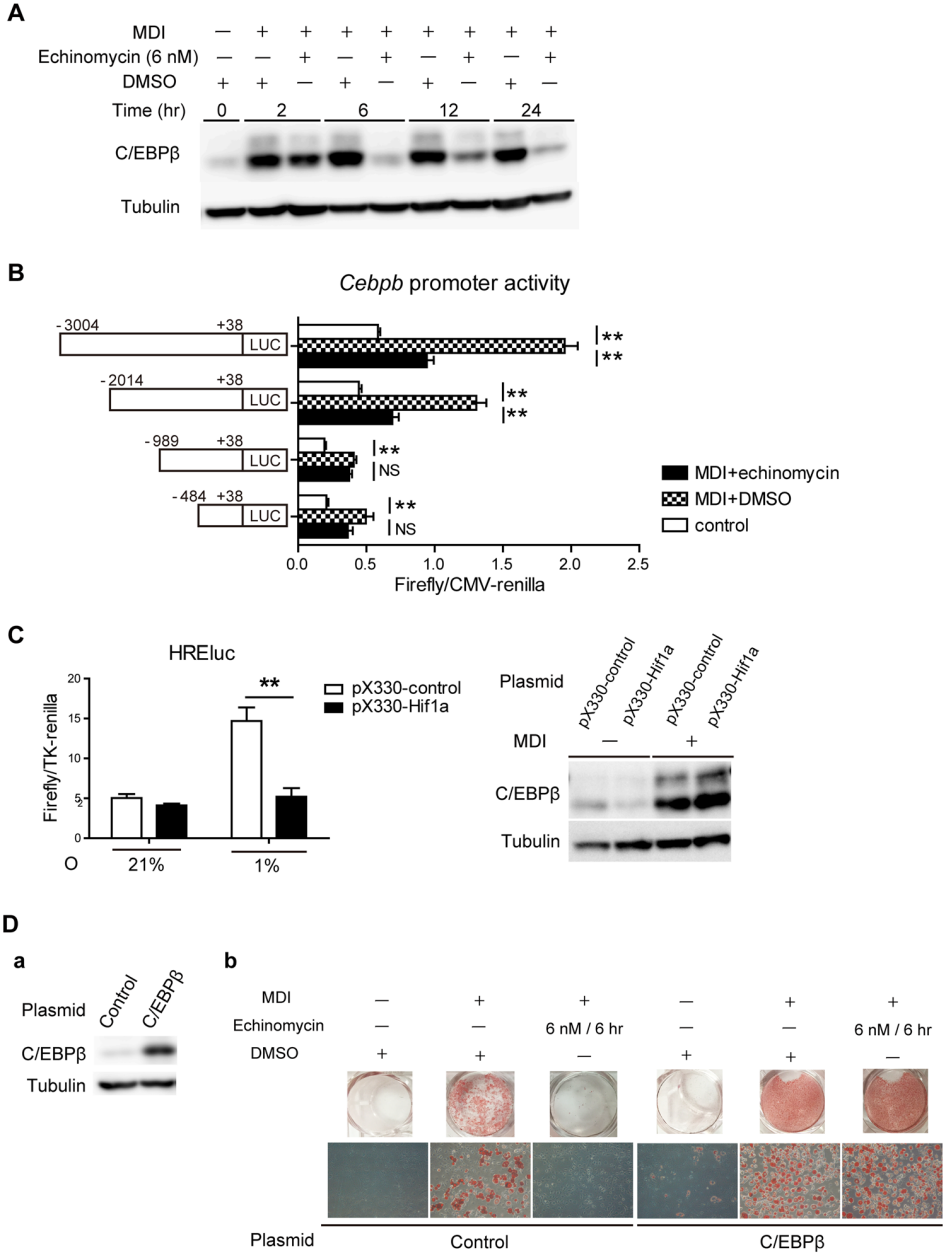
Echinomycin suppresses C/EBPβ expression. (**A**) Immunoblot analysis of C/EBPβ protein in 3T3-L1 cells was performed at 0, 2, 6, 12, and 24 hr after MDI + DMSO or MDI + echinomycin (6 nM) treatment. C/EBPβ protein induction under MDI treatment was inhibited by echinomycin. Full-length blots are presented in Supplementary Figure. (**B**) The activity of the mouse *Cebpb* promoter was measured 8 hr after the treatments. Luciferase activity was normalized to CMV-renilla. *P < 0.05, **P < 0.01 vs. control (only-DMSO treatment), as determined by a two-way ANOVA with Bonferroni’s post-test. (**C**) (*Left panel*) HREluc reporter activity (firefly/TK-renilla) was measured at 16 hr after hypoxia treatment in stably expressing pX-330 against *Hif1a*. **P < 0.01 vs. pX330-control, as determined by a two-way ANOVA with Boneferroni’s post-test. (*Right panel*) Immunoblot analysis of C/EBPβ protein in 3T3-L1 cells that stably expresses pX330-control or pX330-Hif1a at 6 hr after MDI treatment. Knockout of Hif1a by pX330-Hif1a did not influence the induction of C/EBPβ by MDI treatment. Full-length blots are presented in Supplementary Figure. (**D**) (a) Mouse C/EBPβ stable overexpression 3T3-L1 clones were generated using a retroviral system and its C/EBPβ expression was confirmed by immunoblot analysis. Full-length blots are presented in Supplementary Figure. (b) Stable 3T3-L1 clones against control or C/EBPβ were treated with MDI + DMSO or MDI + echinomycin. Oil red O staining was performed at 8 day after induction. C/EBPβ overexpression restored adipogenesis under echinomycin treatment. For all panels, the data are the mean ± SEM or representative of at least three experiments.

Enhanced C/EBPβ protein expression was suppressed by echinomycin continuously from 6 hr after adipogenic induction (Fig. 5A). The result that 2 hr treatment with echinomycin was insufficient to inhibit adipogenesis (Fig. 1B), and the fact that the expression of C/EBPβ in this period is a prerequisite for mitotic clonal expansion in adipogenic differentiation, both support the hypothesis that C/EBPβ is the target. MDI treatment increased the activity of the mouse *Cebpb* promoter, which was significantly decreased by echinomycin treatment, which indicated that echinomycin binds to *Cebpb* promoter and suppresses its transcription (Fig. 5B). To rule out the possibility that C/EBPβ induction by MDI stimuli is dependent on HIF-1, loss-of-function mutations of HIF-1 transcriptional activity was introduced to 3T3-L1 cells through CRISPR/Cas9 system^25^. CRISPR/Cas9-mediated stable knockout 3T3-L1 cells of the *Hif1a* gene was generated by puromycin screening and selecting positive clones using the HREluc assay (Fig. 5C *left panel*). Knockout of *Hif1a* did not suppress the induction of C/EBPβ under MDI stimuli, which indicates that C/EBPβ induction is independent of HIF-1, and that the effect of echinomycin on C/EBPβ is not mediated by HIF-1. In addition, 3T3-L1 cells with retrovirus transduction of C/EBPβ (Fig. 5D-a) escaped from the suppression of adipogenesis by echinomycin (Fig. 5D-b). These results strongly point to C/EBPβ as the main target of echinomycin.

### Echinomycin Inhibits Adipogenesis *in vivo*

**Figure 6:**
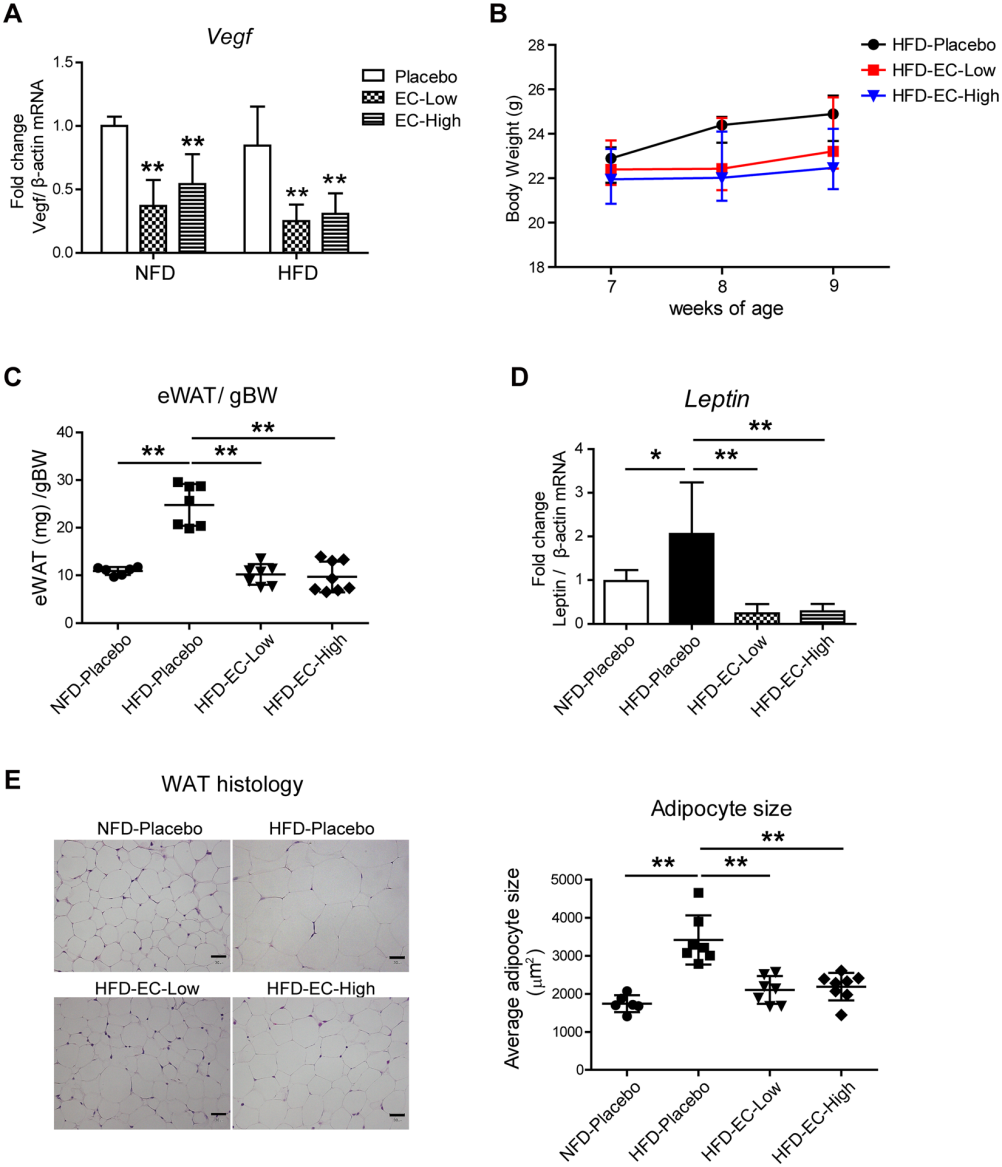
Echinomycin inhibits adipogenesis in high-fat diet mice. (**A**) qRT-PCR in eWAT for a HIF-1 target gene, vascular endothelial growth factor (*Vegf*) in either normal-fat diet (NFD) fed mice or high-fat diet (HFD) fed mice (n = 6–8 in each group). Echinomycin was administered intraperitoneally at two doses and frequency (EC-Low or EC-High). See the Method for further information. **P < 0.01, as determined by a two-way ANOVA with Bonferroni’s post-test. (**B**) Body weight (BW) change in HFD fed mice groups. Echinomycin inhibited the BW gain (n = 6–8). (**C**) eWAT (mg)/BW in each group (n = 6–8). (**D**) qRT-PCR of *leptin* in eWAT (n = 6–8). **P < 0.01, as determined by a two-way ANOVA with Bonferroni’s post-test. (**E**) H&E staining and average size of adipocyte from eWAT (n = 6–8). Scale bar 30 um. **P < 0.01, as determined by a two-way ANOVA with Bonferroni’s post-test.

To further investigate the effect of echinomycin on adipogenesis *in vivo*, male C57BL/6Jcl mice were fed with high-fat diet (HFD) for 2.5 weeks. Echinomycin was intraperitoneally injected at two doses and frequency for the entire period based on the previous reports in mice and humans^26, 27^: 10 μg/kg for 5-days on/2-days off (EC-Low), or 50 μg/kg every alternate day (EC-High). These regimens are much milder than those used in human clinical trials for anti-cancer treatment. Each regimen effectively inhibited the HIF-1 activity (shown by the qRT-PCR for *Vegfa*) in the epididymal white adipose tissue (eWAT) of both normal fat diet (NFD) and HFD mice, which demonstrated that echinomycin was effectively delivered to eWAT (Fig. 6A). Each echinomycin treatment inhibited the body weight gain (Fig. 6B), without effects on the food intake (data not shown). eWAT/gBW and qRT-PCR of the established HFD-induced gene^28^, *leptin*, were analysed among each group, which confirmed that echinomycin inhibited adipogenesis *in vivo* (Fig. 6C and D). H&E staining of eWAT accordingly demonstrated that echinomycin efficiently suppressed the adipocyte size in HFD mice (Fig. 6E). These results are in accordance with the *in vitro* results.

## Discussion

**Figure 7:**
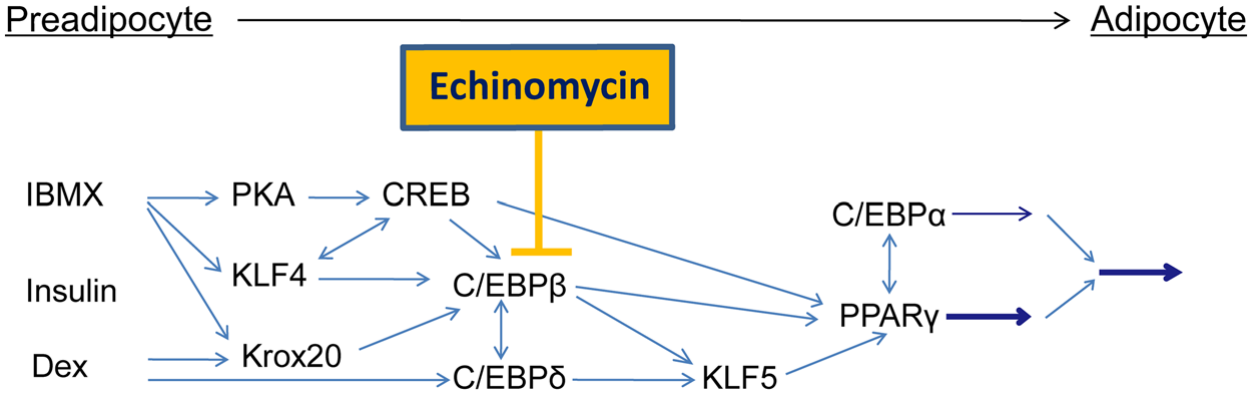
The scheme of echinomycin’s effect on adipogenesis proposed in this study.

Our current study demonstrates that echinomycin, a DNA intercalater, completely inhibits adipogenesis in the 3T3-L1 adipocyte cell line of WAT, as well as in eWAT of HFD mice. A DNA transcriptome array extracted one of the essential adipogenic transcription regulators, C/EBPβ, as its major target (Fig. 7).

An advantage of this small molecule is that a relatively small dose and short-term treatment are sufficient to exhibit its effect. Echinomycin is traditionally known as a HIF-1 inhibitor^7, 8^. It binds to HRE sequences and decreases HIF-1 transcriptional activity. Whether echinomycin affects the expression of HIF-1α protein depends on the cell line; in U251, HepG2, and HeLa cells, HIF1-α protein level is not affected^7, 8, 10^; in MCF-7 cells, it is decreased^11^. In 3T3-L1 cells, echinomycin inhibited HIF-1α protein expression and HIF-1 activity.

The *in vivo* functional role of HIF-1 in adipogenesis of WAT is controversial. Some reported that adipocyte-specific genetic deletion of HIF-1α protects obese mice from insulin resistance and inflammation, whereas constitutively active expression of HIF-1α results in increased insulin resistance and tissue fibrosis^3^. In these knockout mice, white adipose tissue mass and mean adipocyte size are increased^4, 12^, which implies that HIF-1 in the end inhibits adipogenesis in WAT. Others, on the other hand, reported the opposite results using the same *aP2* promoter-driven adipocyte-specific *Hif1a* deleted mice^29, 30^. HIF-1 deficiency improved HFD-induced insulin resistance and reduced WAT adipocity. *In vitro* studies using 3T3-L1 cells show that hypoxia inhibits adipogenesis through HIF-1^12, 14^. Our results also demonstrate that while HIF-1α is induced as early as 2 hr and again from 12 hr after adipogenic induction (data not shown), adipogenesis is suppressed under hypoxia. These findings suggest that although the temporal induction of HIF-1 in the early course of adipogenesis may have some functional roles in regulating the growth arrest and differentiation of preadipocytes, it acts as an inhibitory signal for adipogenesis. Of note, while HIF-2α is known to promote adipogenesis in 3T3-L1 cells, previous reports suggest that HIF-2α is induced at later phase of adipogenesis, from 4 day after MDI induction^12, 13^. We confirmed that HIF-2α was not induced at least until 24 hr after MDI induction (data not shown), and that the inhibitory effect of echinomycin on adipogenesis was unlikely to be mediated by HIF-2.

In contrast to our original expectation, our results demonstrated that echinomycin exerted its inhibitory effect on adipogenesis independently of the HIF pathway. Adipocyte differentiation is precisely controlled by a complex network of transcription factors, cofactors and signalling molecules. A comprehensive DNA microarray analysis was performed, and C/EBPβ was identified as a key target of echinomycin in the adipogenic cascade. C/EBPβ is known as an indispensable adipogenic transcription factor, and ectopic overexpression of C/EBPβ is sufficient to induce PPARγ and subsequent adipocyte differentiation. Continuous suppression of C/EBPβ was observed both at mRNA and the protein level, while C/EBPδ remained unaffected. An *in silico* search predicted several HRE sequences in the promoter region of *Cebpb*, which are potential binding sites for echinomycin, and the loss of this echinomycin effect in *Cebpb* promoters smaller than 1 kbp suggests that echinomycin inhibits the *Cebpb* transcription and its binding site exists between 2 k and 1 k in the promoter region. None of their mutations, however, cancelled the effect of echinomycin on the promoter activity (data not shown), which prevented us from drawing an unequivocal conclusion on its binding site. These observations have two implications for the mechanism of action of echinomycin on C/EBPβ; Echinomycin binds to a *Cebpb* promoter region which is yet to be identified, or, echinomycin binds to an upstream gene of *Cebpb*, which results in the transcriptional inhibition of C/EBPβ. Although our microarray results and *Cebpb* promoter assay favour the former story, the exact mechanism of action of echinomycin on C/EBPβ remains to be a subject of future research.

There are some other reports on inhibitory molecules against adipogenesis. Rapamycin, an inhibitor of mammalian target of rapamycin, suppresses adipogenic differentiation through its anti-proliferative activity^31^. Pyridinyl imidazoles also block 3T3-L1 adipogenesis by targeting p38 mitogen-activated protein kinase^32^. Our study gives an additional insight into how adipogenesis could be blocked by a temporal intervention. It is also unique that it affects the very early phase of transcriptional cascade.

Taken together, our results demonstrate a novel effect of echinomycin beyond its anti-tumour activity, by acting on adipocyte differentiation. Echinomycin inhibits adipogenesis of 3T3-L1 cells in a HIF-independent manner. Time-course DNA microarray analysis of drug-treated and untreated preadipocytes extracted a major transcription factor, CCAAT/enhancer-protein β, as a key target of echinomycin. The effect of echinomycin also applied to eWAT of experimental obesity model, HFD mice. This study provides further molecular insight into the spatially and temporally regulated cascade of WAT adipocytes.

## Materials and Methods

### Reagents

Insulin, dexamethasone (DEX), 3-isobutyl-1-methylxanthine (IBMX), and oil red O were purchased from Sigma (Sigma-Aldrich, St Louis, MO). Echinomycin (Calbiochem, Gibbstown, NJ) was dissolved in DMSO (Wako, Tokyo, Japan) according to the manufacturer’s instructions.

### Cell Culture, Differentiation, and Drug Treatment

3T3-L1 cells (American Type Culture Collection, Rockville, MD) were cultured in Dulbecco’s modified Eagle’s medium (DMEM, Nissui, Tokyo, Japan) supplemented with 10% calf serum (CS). The differentiation protocol is as described previously^33^. Briefly, two days after cells reached confluence (Day 0), the medium was replaced with a mixture consisting of 10% FCS and MDI (0. 5 mM IBMX, 1 μM DEX, and 10 μg/ml insulin). After 48 h, the medium was changed to DMEM containing 10% FCS and 10 μg/ml insulin. The medium was replenished at 2-day intervals, and the appearance of cytoplasmic triglycerides was monitored by microscopy and confirmed by staining with oil red O. For hypoxic stimulation, cells were exposed to 1% O_2_/5% CO_2_, with nitrogen as the balance, in a multigas incubator, APM-30D (ASTEC, Fukuoka, Japan).

### Oil Red O Staining

At day 8, 3T3-L1 cells were rinsed with PBS twice and fixed with 10% formaldehyde in H_2_O for 10 min. The cells were treated with 60% isopropanol in H_2_O for 1 min and then stained in freshly diluted oil red O solution (0.18% (w/v) oil red O in 60% isopropanol) for 20–40 mins until the cells were stained. The cells were then washed with 60% isopropanol in H_2_O and twice with PBS. The cells were observed in PBS under a microscope and photographed. Oil red O quantification was performed by extracting the dye by 100% isopropanol and OD 490 nm was subsequently measured in 96-well dishes.

### Luciferase Reporter Assay

HIF-1 transcriptional activity (HREluc activity) was measured by dual-luciferase reporter assays (Promega, Madison, WI) in 24-well culture dishes. Five-hundred nanograms of pGL3 (Promega) vector driven by 7× hypoxia-responsive elements (pHREluc)^34^ and 25 ng of *Renilla* luciferase vector (pRL-TK or pRL-CMV) were co-transfected using Lipofectamine LTX (Invitrogen, Carlsbad, CA); 2 days after full confluence, the cells were differentiated as above and exposed to hypoxia (1% O_2_) for 8 hr. The cells were processed in fixed protein aliquots, and HREluc activity was measured using a Lumat 9507 luminometer (EG and Berthold, Bad Wildbad, Germany). The relative light unit (RLU) value of firefly luciferase was divided by that of *Renilla* luciferase to correct for the transfection efficiency. To measure *Cebpb* promoter activity, a similar method to the above was used. *Cebpb* promoter fragments of 3 kb, 2 kb, 1 kb, or 0.5 kb (mCEBPB3K, 2 K, 1 K, or 0.5 K promoter) were PCR amplified from mouse genomic DNA using PrimeStar HS DNA polymerase (Takara Bio, Shiga, Japan) and primers with *KpnI* and *XhoI* overhangs (see Table 1 for primers). Digested and purified fragments were inserted into the pGL3basic reporter vector (Promega).

**Table 1:** A list of primers used in this study.

Gene	use	species	F/R	Sequence
*Actb*	RT-PCR	mouse	Forward	CTTTCTACAATGAGCTGCGTG
			Reverse	TCATGAGGTAGTCTGTCAGG
*Ap2* (*Fabp4*)	RT-PCR	mouse	Forward	AACACCGAGATTTCCTT
			Reverse	ACACATTCCACCACCAG
*Cebpa*	RT-PCR	mouse	Forward	GCCTTCAACGACGAGTTCCT
			Reverse	CTCCCGGGTAGTCAAAGTCA
*Cebpb*	RT-PCR	mouse	Forward	GCAAGAGCCGCGACAAG
			Reverse	GGCTCGGGCAGCTGCTT
*Cebpd*	RT-PCR	mouse	Forward	TCGACTTCAGCGCCTACATTGACT
			Reverse	CCGCTTTGTGGTTGCTGTTGAAGA
*Glut1*	RT-PCR	mouse	Forward	CAGTTCGGCTATAACACCGGTGTC
			Reverse	ATAGCGGTGGTTCCATGTTT
*Hk2*	RT-PCR	mouse	Forward	CAACATCCTGATCGATTTCACA
			Reverse	GCAGTCACTCTCGATCTGAGAC
*Klf4*	RT-PCR	mouse	Forward	GCGGGAAGGGAGAAGACAC
			Reverse	GGCTCGGGCAGCTGCTT
*Klf5*	RT-PCR	mouse	Forward	GGTTGCACAAAAGTTTATAC
			Reverse	GGCTTGGCGCCCGTGTGCTTCC
*Pparg*	RT-PCR	mouse	Forward	CCAGGCTTGCTGAACGTGAA
			Reverse	GGGGAAGACGAGGATGAAGC
*Vegf*	RT-PCR	mouse	Forward	TTACTGCTGTACCTCCAC
			Reverse	ACAGGACGGCTTGAAGATA
*leptin*	RT-PCR	mouse	Forward	GTGGTCGGAAGCCCTGAGATAG
			Reverse	GGGCGATCACTCGATGGAA
gRNA_mHif1a437	gRNA	mouse	Forward	CACCGTTTCTTCTCGTTCTCGCCGC
			Reverse	AAACGCGGCGAGAACGAGAAGAAAC
gRNA_mHif1a940	gRNA	mouse	Forward	CACCGAAGTGCACCCTAACAAGCCG
			Reverse	AAACCGGCTTGTTAGGGTGCACTTC
mCEBPB3k	Reporter assay	mouse	Forward	GGCGGTACCTTAGCAACCATCACAGCCACAG
			Reverse	GCCTCGAGCGGGAGGTTTATAAGGC
mCEBPB2k	Reporter assay	mouse	Forward	GGCGGTACCTGTGATCGCAAACAGT
			Reverse	GCCTCGAGCGGGAGGTTTATAAGGC
mCEBPB1k	Reporter assay	mouse	Forward	GCCGGTACCTGACAGGTTGGCAGCT
			Reverse	GCCTCGAGCGGGAGGTTTATAAGGC
mCEBPB0.5k	Reporter assay	mouse	Forward	GGCGGTACCTGGAGAGTTCTGCTTC
			Reverse	GCCTCGAGCGGGAGGTTTATAAGGC

### Plasmids and Stable Overexpression

3T3-L1 clones, which stably overexpress C/EBPβ, were generated with retrovirus transduction (Platinum Retrovirus Expression System, Pantropic, Cell Biolabs, San Diego, CA). pcDNA-mC/EBPb was a gift from Jed Friedman (Addgene plasmid #49198). The plasmid was digested with *XhoI* and *PmeI*, and subcloned into the retroviral vector, pMXs-IRES-Puro using the *XhoI*-*SnaBI* site. The plasmids were transfected to the packaging cell line, and the culture supernatants were collected 24 and 48 hr later, passed through 0.22-μm filters, and added to the cells. Drug selection by 2 μg/ml puromycin was initiated 72 hr after infection. For CRISPR-Cas9 system, pX330-U6-Chimeric_BB-CBh-hSpCas9 was a gift from Feng Zhang (Addgene plasmid #42230)^24^. Hif1a target sgRNAs were designed by using CRISPRdirect^35^, and ligated into pX330 plasmid by following the procedures from Zhang lab (http://www.genome-engineering.org/crispr). Sequences are listed in Table 1. 3T3-L1 cells were co-transfected with the two pX330-Hif1a and a puromycin resistant plasmid which facilitates selection with puromycin (2 μg/ml). Selected colonies went further selection by HREluc activity under hypoxia.

### Microarray Analysis

A transcriptome microarray analysis of 3T3-L1 cells was conducted at 0 hr, 2 hr, 6 hr, and 24 hr of treatment with either MDI plus DMSO or MDI plus echinomycin (6 nM). For the 24-hr samples, cells were washed and re-incubated with MDI 6 hr after induction. Total cellular RNA was isolated using RNAiso plus (Takara Bio). cRNA was synthesized from cellular RNA and hybridized to high density Affymetrix microarray gene chips containing 45,102 probe sets. The expression value for each mRNA was obtained using the Robust Multi-array Analysis (RMA) method. Probes with normalized intensity lower than 100 in all seven arrays (31,097 probes) were excluded from the analyses, which left 14,005 probes for further analysis. To identify the transcripts that were increased by MDI treatment, all probes with a fold change >= 2.0 in expression (either 2, 6, or 24 hr after MDI + DMSO treatment) compared with the control (time 0 hr) were selected (2,204 probes), and hierarchical clustering analysis was performed using the average linkage and relative correlation as a measure of similarity for the selected genes. To identify transcripts that are significantly induced by MDI and suppressed both at 2 hr and 6 hr after echinomycin treatment, the following selection procedures were performed. All probes that had a fold change >= 2.0 in expression compared with the control (time 0 hr) at both two time points, 2 hr and 6 hr, were first selected (592 probes). After excluding probe sets that did not have gene annotation, the remaining 564 probes were used for further analyses. Hierarchical clustering analysis of a log 2-fold change from the control (time 0 hr) was performed using the average linkage and relative correlation as a measure of similarity for the selected genes. The Database for Annotation, Visualization and Integrated Discovery (DAVID)^36, 37^, an online gene database provided by the NIH (version 6.8; http://david.abcc.ncifcrf.gov/), was used to investigate biological functions associated with the gene lists.

### RNA Isolation and Real-Time Quantitative RT-PCR (qRT-PCR)

RNA isolation and real-time quantitative (q) RT-PCR assays were performed as previously described^38^. The data were calibrated to the β-actin value. The primers are described in Table 1.

### Immunoblotting Analysis

The cells were collected for immunoblotting analysis as previously described^38^. The primary antibodies were as follows: anti-HIF1α (Novus Biologicals, Littleton, CO); anti-HIF2α (Novus Biologicals, Littleton, CO); anti-GLUT1 (Abcam plc, Cambridge, UK); anti-C/EBPβ (Santa Cruz Biotechnology, Santa Cruz, CA); and anti-α-tubulin (Cell Signaling Technology, Danvers, MA). HRP-conjugated anti-rabbit IgG (Bio-Rad Laboratories, Hercules CA) or anti-mouse IgG (Bio-Rad Laboratories) antibodies were used as secondary antibodies. The signals were detected with the ECL Plus reagent (Thermo Fischer Scientific, Waltham, MA) using the chemiluminescence protocol. For detecting the signals of GLUT1 and HIF-2α, SuperSignal West Femto Maximum Sensitivity Substrate (Thermo Fischer Scientific) was used.

### Animal Experiments

Male C57BL/6NJcl mice aged 7 weeks (Nippon Seibutsu Zairyo Center, Tokyo, Japan) were fed with either normal fat diet or high fat diet (HFD 60) containing 60% of fat (Oriental Yeas Co. Ltd., Tokyo, Japan). Echinomycin was dissolved in DMSO at 5 mg/ml and subsequently diluted with 10 mg/ml BSA/saline to a concentration of 0.0293 mg/ml. Either 1)10 μg/kg for 5-days on/2-days off, or, 2) 50 ug/kg alternate day, was intraperitoneally injected until the day of sacrifice. At sacrifice, the mice were euthanized and the epididymal white adipose tissue (eWAT) were harvested, measured, fixed in formalin for immunohistochemical analysis, snap-frozen in OCT, or stored at −80 °C for qRT-PCR analysis. All mice were housed in the animal care facility of the University of Tokyo under standardized conditions (25 °C, 50% humidity, 12 hr light/dark cycle) with food and water available at libitum. All the experiments were carried out in accordance with the guidelines and regulations of the Committee on Ethical Animal Care and Use at the University of Tokyo Graduate School of Medicine, as well as with the guidelines of the National Institutes of Health for the use of animals in research. All experimental protocols were approved by the animal care and use committee at the University of Tokyo Graduate School of Medicine (Approval Number: M-P16-104). For adipocyte size measurement, eWAT was processed for paraffin embedding, and 3-μm tissue sections were stained with hematoxylin and eosin. The adipocyte areas were manually traced and analyzed using the Image J software.

### Statistical Analysis

All data are reported as the mean ± SEM or the mean ± SD. The data for two groups were analysed using Student’s unpaired *t* test. The differences among more than two groups were analysed using one-way ANOVA with Tukey’s post-test or two-way ANOVA with Bonferroni’s post-test. Differences with a *P* value < 0.05 were considered significant. GraphPad Prism version 5.04 for Windows (GraphPad Software, San Diego, CA) was used for data analysis.

## Electronic supplementary material

Supplementary Information
