# Supplementary material for: Echinomycin inhibits adipogenesis in 3T3-L1 cells in a HIF-independent manner
Source: Sci Rep. 2017 Jul 26;7:6516. doi: 10.1038/s41598-017-06761-4 (PMC5529514; doi:10.1038/s41598-017-06761-4)
Supplement: Supplementary file 1 — Supplementary Information [file 41598_2017_6761_MOESM1_ESM.pdf]

Echinomycin inhibits adipogenesis in 3T3-L1 cells in a HIF-independent manner
Junna Yamaguchi, Tetsuhiro Tanaka, Hisako Saito, Seitaro Nomura, Hiroyuki Aburatani, Hironori Waki, Takashi Kadowaki, Masaomi Nangaku

Table S1. The list of 2,204 probes in the microarray analysis

Cluster 1 (751 probes)		Cluster 2 (907probes)		Cluster 3 (546probes)	
**Probe Set ID**	**Gene Symbol**	**Probe Set ID**	**Gene Symbol**	**Probe Set ID**	**Gene Symbol**
1417483_at	Nfkbiz	1451666_at	Acly	1416101_a_at	Hist1h1c
1416630_at	Id3	1426533_at	Nop56	1436994_a_at	Hist1h1c
1418932_at	Nfil3	1427504_s_at	Srsf2	1419022_a_at	Eno1
1435162_at	Prkg2	1435335_a_at	Gnptab	1427404_x_at	Gm5506
1424289_at	Osgin2	1433738_at	Papd5	1420478_at	Nap1l1
1415899_at	Junb	1416034_at	Cd24a	1416479_a_at	Tmem14c
1426223_at	Ttc39c	1435945_a_at	Kcnn4	1452927_x_at	Tpi1
1440041_at	---	1448270_at	Ddx21	1448754_at	Rbp1
1425503_at	Gcnt2	1452640_at	3110007F17Rik	1416708_a_at	Gramd1a
1423233_at	Cebpd	1422767_at	Bysl	1434799_x_at	Aldoa
1426418_at	Atoh8	1426394_at	Eif3j	1439795_at	Gpr64
1439348_at	S100a10	1450084_s_at	Ivns1abp	1415918_a_at	Tpi1
1425895_a_at	Id1	1424500_at	Utp6	1416335_at	Mif
1424942_a_at	Myc	1452052_s_at	Eif3j	1444952_a_at	Nucks1
1434025_at	---	1460541_at	Slc7a6	1451967_x_at	Kpnb1
1456816_at	---	1423703_at	Ppan	1454783_at	Il13ra1
1448830_at	Dusp1	1420401_a_at	Ramp3	1452296_at	Slit3
1447314_at	---	1448319_at	Akr1b3	1452190_at	Prcp
1429053_at	1110012J17Rik	1418300_a_at	Mknk2	1460353_at	Tmem48
1418901_at	Cebpb	1437133_x_at	Akr1b3	1450826_a_at	Saa3
1448793_a_at	Sdc4	1460551_at	Ran	1452681_at	Dtymk
1442608_at	Layn	1455904_at	Gas5	1448441_at	Cks1b
1451177_at	Dnajb4	1456117_at	Rrp1b	1423666_s_at	LOC100503670
1451021_a_at	Klf5	1416529_at	Emp1	1427921_s_at	2310061C15Rik
1448231_at	Fkbp5	1424574_at	Tmed5	1419823_s_at	Ksr1
1423267_s_at	Itga5	1417406_at	Sertad1	1431054_at	Lsm6
1416125_at	Fkbp5	1416545_at	Zdhhc7	1438096_a_at	Dtymk
1417871_at	Hsd17b7	1431789_s_at	Tmed5	1426002_a_at	Cdc7
1427844_a_at	Cebpb	1424573_at	Tmed5	1455892_x_at	---
1434384_at	Nrip1	1437502_x_at	Cd24a	1425789_s_at	Anxa8
1427144_at	Hnrpll	1455035_s_at	Nop56	1424006_at	Aarsd1
1455899_x_at	Socs3	1458268_s_at	Igfbp3	1416698_a_at	Cks1b
1444107_at	C130039O16Rik	1428950_s_at	Nol8	1440715_s_at	Cdkn2aipnl
1416505_at	Nr4a1	1433502_s_at	Tsr1	1448975_s_at	Ren1 /// Ren2
1448742_at	Snai1	1455643_s_at	Tsr1	1418026_at	Exo1
1449851_at	Per1	1434398_at	Nkrf	1437181_at	Peli2
1457248_x_at	Hsd17b7	1448182_a_at	Cd24a	1420477_at	Nap1l1
1428393_at	Nrn1	1448271_a_at	Ddx21	1446656_at	---
1455881_at	Ier5l	1419912_s_at	Strap	1452200_at	Cdkn2aipnl
1449037_at	Crem	1441272_at	Matr3	1423785_at	Egln1
1442213_at	LOC552908	1425610_s_at	Galnt2	1433807_at	6720463M24Rik
1426812_a_at	Fam129b	1421876_at	Mapk9	1415860_at	Kpna2
1424296_at	Gclc	1426236_a_at	Glul	1449116_a_at	Dtymk
1431734_a_at	Dnajb4	1450846_at	Bzw1	1435639_at	2610528A11Rik
1455959_s_at	Gclc	1427820_at	---	1435659_a_at	Tpi1
1451739_at	Klf5	1424883_s_at	Srsf7	1449648_s_at	Polr1c
1417487_at	Fosl1	1420772_a_at	Tsc22d3	1423211_at	Nop10
1428942_at	Mt2	1415907_at	Ccnd3	1415829_at	Lbr
1416743_at	LOC640502	1426235_a_at	Glul	1422327_s_at	G6pd2
1415834_at	Dusp6	1423039_a_at	Bzw1	1415857_at	Emb
1437658_a_at	Snhg1	1428296_at	Gm10774	1417732_at	Anxa8

1431182_at	Hspa8	1453745_at	2700038G22Rik	1416553_at	Stra13
1459305_at	---	1452096_s_at	D230025D16Rik	1426612_at	Tipin
1418176_at	Vdr	1426645_at	Hsp90aa1	1448354_at	G6pdx
1416442_at	Ier2	1448204_at	Sav1	1420592_a_at	Anp32e
1422751_at	Tle1	1439615_at	Gan	1439148_a_at	Pfkl
1418753_at	Gfpt2	1430980_a_at	Eif4a1	1418777_at	Ccl25
1460206_at	Grasp	1419385_a_at	Ubqln1	1450269_a_at	Pfkl
1423474_at	Top1	1418507_s_at	Socs2	1434695_at	Dtl
1420817_at	Ywhag	1425281_a_at	Tsc22d3	1458648_at	AU042950
1415913_at	Gm12270	1452490_a_at	Ap2a2	1452406_x_at	Erdr1
1418469_at	Nrip1	1456071_a_at	Cycs	1436737_a_at	Sorbs1
1426825_at	Fmnl3	1422801_at	G3bp1	1428471_at	Sorbs1
1418175_at	Vdr	1428090_at	Ptcd3	1419737_a_at	Ldha
1438739_at	---	1422483_a_at	Cycs	1417566_at	Abhd5
1420548_a_at	2310008H09Rik	1451006_at	Xdh	1425780_a_at	Tmem167
1438862_at	---	1448250_at	9030425E11Rik	1435749_at	Gda
1423903_at	Pvr	1422667_at	Krt15	1421694_a_at	Vcan
1422452_at	Bag3	1452461_a_at	Gnptab	1427747_a_at	Lcn2
1437247_at	Fosl2	1425326_at	Acly	1415998_at	Vdac1
1417372_a_at	Peli1	1435544_at	Aars	1419603_at	Ifi204
1425837_a_at	Ccrn4l	1433789_at	Snhg3	1426554_a_at	Pgam1
1425742_a_at	Tsc22d1	1439027_at	Naa25	1416164_at	Fbln5
1434680_at	Plekhg3	1425656_a_at	Baiap2	1423947_at	1110008P14Rik
1418835_at	Phlda1	1429587_at	Rbm34	1435748_at	Gda
1435039_a_at	Pip5k1a	1460260_s_at	Kpna1	1433675_at	Snhg1
1417654_at	Sdc4	1422444_at	Itga6	1434329_s_at	Adipor2
1456212_x_at	Socs3	1455988_a_at	Cct6a	1452094_at	P4ha1
1454971_x_at	Tsc22d1	1423517_at	Cct6a	1448613_at	Ecm1
1416576_at	Socs3	1426351_at	Hspd1	1433674_a_at	Snhg1
1443088_at	9930031P18Rik	1424843_a_at	Gas5	1415856_at	Emb
1416129_at	Errfi1	1430147_a_at	Taf1d	1416119_at	Txn1
1435248_a_at	Btaf1	1435740_at	Gm10397	1416249_at	Nadk
1422537_a_at	Id2	1450986_at	Nop58	1424860_at	D930016D06Rik
1416431_at	Tubb6	1452168_x_at	Gspt1	1438073_at	Spry3
1436871_at	Srsf7	1448484_at	Amd1	1426947_x_at	Col6a2
1419816_s_at	Errfi1	1450881_s_at	Gpr137b	1452348_s_at	Ifi204
1448950_at	Il1r1	1428135_a_at	Eef1d	1442884_at	Hgf
1441315_s_at	Slc19a2	1451012_a_at	Csda	1429478_at	6720463M24Rik
1449110_at	Rhob	1450782_at	Wnt4	1417864_at	Pgk1
1437696_at	BC049807	1434242_at	Usp37	1439618_at	Pde10a
1434303_at	Raph1	1441687_at	LOC100503611	1417565_at	Abhd5
1455657_at	Smg1	1422788_at	Slc43a3	1425826_a_at	Sorbs1
1419069_at	Rabgef1	1425836_a_at	Limk1	1451775_s_at	Il13ra1
1423667_at	Mat2a	1418527_a_at	Srsf10	1423210_a_at	Nop10
1424246_a_at	Tes	1452213_at	Tex2	1420981_a_at	Lmo4
1420909_at	Vegfa	1417627_a_at	Limk1	1428776_at	Slc10a6
1451717_s_at	Senp2	1449109_at	Socs2	1460238_at	Msln
1418302_at	Ppt2	1415910_s_at	Ciapin1	1451171_at	2310008H04Rik
1452519_a_at	Zfp36	1454606_at	4933426M11Rik	1427164_at	Il13ra1
1454758_a_at	Tsc22d1	1421877_at	Mapk9	1449212_at	Pip
1450750_a_at	Nr4a2	1417032_at	Ube2g2	1448666_s_at	Tob2
1447863_s_at	Nr4a2	1453849_s_at	Hnrnpab	1443983_at	---
1455034_at	Nr4a2	1428275_at	Abhd13	1444472_at	---
1425466_at	Senp2	1422445_at	Itga6	1429590_at	Tacc1
1425465_a_at	Senp2	1437738_at	Atp2c1	1451866_a_at	Hgf
1438796_at	Nr4a3	1422454_at	Krt13	1450387_s_at	Ak4
1434261_at	Sipa1l2	1423062_at	Igfbp3	1424854_at	Hist1h4a
1419485_at	Foxc1	1434202_a_at	Fam107a	1448601_s_at	Msx1

1416029_at	Klf10	1460036_at	Ap1s2	1421830_at	Ak4
1428562_at	2210403K04Rik	1435800_a_at	Csda	1440311_at	Sorbs1
1451819_at	Zswim6	1425930_a_at	Mlx	1415852_at	Impdh2
1438495_at	Top1	1452662_a_at	Eif2s1	1434278_at	Mtm1
1451208_at	Etf1	1438945_x_at	Gja1	1415851_a_at	Impdh2
1435249_at	Btaf1	1444361_at	---	1454935_at	Fitm2
1417902_at	Slc19a2	1422484_at	Cycs	1428593_at	1700029F09Rik
1459722_at	Zswim6	1437007_x_at	Usp39	1416871_at	Adam8
1449449_at	Ptges	1438546_x_at	Slc25a5	1426992_at	Xpr1
1438019_at	Ippk	1456620_at	Gnptab	1423827_s_at	Noc4l
1443969_at	Irs2	1424474_a_at	Camkk2	1417972_s_at	Pop5
1428433_at	Hipk2	1454957_at	Nob1	1454109_a_at	Jmjd6
1434967_at	Zswim6	1436000_a_at	Skp2	1423708_a_at	Farsb
1458469_at	Cblb	1450423_s_at	Rpf2	1439435_x_at	Pgk1
1430697_at	Ammecr1	1423795_at	Sfpq	1448809_at	Cse1l
1435176_a_at	Id2	1418225_at	Orc2	1416439_at	Dctpp1
1422168_a_at	Bdnf	1428277_at	Otud6b	1449198_a_at	St3gal5
1449089_at	Nrip1	1425718_a_at	Ivns1abp	1416850_s_at	Cisd1
1419149_at	Serpine1	1435695_a_at	Ggct	1417353_x_at	Snrpa1
1444165_at	Layn	1428553_at	Glrx5	1436966_at	Peli2
1451959_a_at	Vegfa	1456738_s_at	Brp16	1423749_s_at	Rangap1
1429321_at	Rnf149	1429169_at	Rbm3	1451385_at	Fam162a
1454724_x_at	Fam108b	1428542_at	Eif1ad	1417308_at	Pkm2
1456477_at	Ccnt1	1447209_at	---	1417352_s_at	Snrpa1
1442332_at	---	1427256_at	Vcan	1417149_at	P4ha2
1419431_at	Ereg	1416218_x_at	Rpl37a	1434509_at	Rapgef6
1443394_at	---	1455345_at	Phf15	1420056_s_at	Jmjd6
1427348_at	Zc3h12a	1438527_at	Gm5879	1436796_at	LOC100503380
1433771_at	Fam108b	1416503_at	Lxn	1451092_a_at	Rangap1
1419766_at	Sik1	1425553_s_at	Hip1r	1430353_at	Glis3
1440643_at	---	1438458_a_at	Sfpq	1416042_s_at	Nasp
1438187_at	Slc25a29	1439399_a_at	Snhg1	1453183_at	1110034A24Rik
1422702_at	Azin1	1451071_a_at	Atp1a1	1436544_at	Atp10d
1458241_at	---	1441070_at	LOC100504154	1447999_x_at	Gapdh
1441823_at	Zmiz1	1422411_s_at	BC151093	1451204_at	Scara5
1416502_a_at	Preb	1425552_at	Hip1r	1426529_a_at	Tagln2
1417621_at	Nfatc1	1416726_s_at	Ube2s	1417480_at	Fbxo9
1451714_a_at	Map2k3	1423810_at	Ppme1	1452661_at	Tfrc
1455342_at	Prune2	1416075_at	Sav1	1417109_at	Tinagl1
1437239_x_at	Phc2	1450904_at	Tmem167	1415909_at	Stip1
1421321_a_at	Net1	1423208_at	Tmem167	1436705_at	Mmgt1
1435644_at	Sh3pxd2b	1415850_at	Rasa3	1428589_at	Mrpl41
1428902_at	Chst11	1416120_at	Rrm2	1448140_at	Ciapin1
1427299_at	Rps6ka3	1443253_at	---	1452608_at	Mycbp
1452359_at	Rell1	1437923_at	AI314760	1423863_at	Abcf2
1419656_at	Slc25a36	1420810_at	1500003O03Rik	1423357_at	Lipt2
1427243_at	Rell1	1437210_a_at	Brd2	1418674_at	Osmr
1458616_at	---	1417309_at	Tob2	1427257_at	Vcan
1454755_at	Itpkc	1422474_at	Pde4b	1416480_a_at	Gm9790
1418099_at	Tnfrsf1b	1418879_at	Fam110c	1431997_at	3000002C10Rik
1424013_at	Etf1	1423585_at	Igfbp7	1416746_at	H2afx
1425456_a_at	Map2k3	1448226_at	Rrm2	1437045_at	Mapk8
1433724_at	D15Ertd621e	1434437_x_at	Rrm2	1450743_s_at	Syncrip
1437493_at	5230400M03Rik	1428132_at	Cdc42se1	1415901_at	Plod3
1417162_at	Tmbim1	1448377_at	Slpi	1423653_at	Atp1a1
1430352_at	Adamts9	1449824_at	Prg4	1428131_a_at	Cdc42se1
1450749_a_at	Nr4a2	1448667_x_at	Tob2	1416481_s_at	Higd1a
1438133_a_at	Cyr61	1449336_a_at	Slk	1435474_at	Taf5

1416039_x_at	Cyr61	1422473_at	Pde4b	1424110_a_at	Nme1
1435777_at	Itpripl2	1453906_at	Med13l	1436181_at	Asap2
1451415_at	1810011O10Rik	1421354_at	Prkg2	1423642_at	Tubb2c
1454788_at	Arl4c	1457644_s_at	Cxcl1	1452743_at	Pole3
1436512_at	Arl4c	1448503_at	Mcl1	1447930_at	Baz1a
1449168_a_at	Akap2	1451982_at	Map2k4	1416073_a_at	Nup85
1440332_at	Cdv3	1420150_at	Spsb1	1429057_at	Naa16
1439797_at	Ppard	1441894_s_at	Grasp	1427760_s_at	Prl2c2
1427929_a_at	Pdxk	1448865_at	Hsd17b7	1416203_at	Aqp1
1460510_a_at	Coq10b	1435655_at	Rpl12	1435167_at	Ranbp6
1422560_at	Ddi2	1423766_at	Pak1ip1	1417351_a_at	Snrpa1
1438657_x_at	Gm13363	1416067_at	Ifrd1	1424348_at	1110007A13Rik
1433943_at	Itprip	1418946_at	St3gal1	1434754_at	Rap1gap2
1427931_s_at	Pdxk	1451532_s_at	Steap1	1422412_x_at	Ear3
1416701_at	Rnd3	1424938_at	Steap1	1418659_at	Clock
1426721_s_at	Tiparp	1434089_at	Synpo	1451527_at	Pcolce2
1433576_at	Mat2a	1451272_a_at	Ube2f	1431125_a_at	Tars2
1425351_at	Srxn1	1449450_at	Ptges	1427938_at	Mycbp
1451027_at	Baiap2	1434312_at	Arf6	1416433_at	Rpa2
1422864_at	Runx1	1430034_at	Cct4	1433482_a_at	Fubp1
1447411_at	---	1416308_at	Ugdh	1424143_a_at	Cdt1
1449322_at	Gm13363	1457150_at	AI428301	1423924_s_at	Tspan14
1444326_at	---	1435110_at	Unc5b	1417667_a_at	Pter
1419024_at	Ptp4a1	1444538_at	---	1419270_a_at	Dut
1417262_at	Ptgs2	1457944_at	---	1417948_s_at	Ilf2
1442069_at	Ept1	1452119_at	Rrp1b	1417886_at	1810009A15Rik
1417723_at	Ube2j1	1416811_s_at	Ctla2a	1426447_at	Nup35
1419078_at	Nin	1448471_a_at	Ctla2a	1422535_at	Ccne2
1455870_at	Akap2	1416745_x_at	LOC640502	1435114_at	Wdhd1
1454648_s_at	D10Wsu102e	1418250_at	Arl4d	1436708_x_at	Mcm4
1441843_s_at	5230400M03Rik	1434376_at	Cd44	1434683_at	---
1428834_at	Dusp4	1418911_s_at	Acsl4	1452241_at	Topbp1
1451253_at	Pxk	1449901_a_at	Map3k6	1421731_a_at	Fen1
1437743_at	Aebp2	1416880_at	Mcl1	1429395_at	Gstcd
1428487_s_at	Coq10b	1433531_at	Acsl4	1459897_a_at	Sbsn
1427930_at	Pdxk	1460227_at	Timp1	1439651_at	---
1421034_a_at	Il4ra	1437527_x_at	Mcl1	1423218_a_at	Mrpl49
1450714_at	Azin1	1426233_at	Map2k4	1419645_at	Cstf2
1435595_at	1810011O10Rik	1426348_at	Col4a1	1433547_s_at	Nudcd1
1437199_at	Dusp5	1422943_a_at	Hspb1	1436422_at	BC026590
1452160_at	Tiparp	1426370_at	Far1	1426492_at	Tdp1
1433617_s_at	B4galt5	1430875_a_at	Pak1ip1	1417144_at	Tubg1
1452675_at	Rbm22	1416744_at	LOC640502	1439630_x_at	Sbsn
1439168_at	Camk2d	1448232_x_at	Gm5620	1434224_at	Tbl2
1417740_at	Cdc37l1	1450262_at	Clcf1	1452996_a_at	Aven
1453149_at	Slc25a32	1433555_at	Eaf1	1416544_at	Ezh2
1418102_at	Hes1	1438405_at	Fgf7	1457827_at	Arsj
1434302_at	LOC100504603	1417430_at	Cdr2	1424300_at	Gemin6
1427763_a_at	Camk2d	1426337_a_at	Tead4	1417586_at	Timeless
1437742_at	Rab21	1440120_at	---	1427382_a_at	Suv39h1
1452161_at	Tiparp	1456381_x_at	Mcl1	1454011_a_at	Rpa2
1458802_at	Hivep3	1436155_at	Nmnat2	1416030_a_at	Mcm7
1456341_a_at	Klf9	1449002_at	Phlda3	1423809_at	Tcf19
1416700_at	Rnd3	1452182_at	Galnt2	1424632_a_at	Rev3l
1421624_a_at	Enah	1449268_at	Gfpt1	1449530_at	Trps1
1419357_at	Isy1	1425658_at	Cd109	1439436_x_at	Incenp
1459865_x_at	Ces5a	1419682_a_at	2810408M09Rik	1415878_at	Rrm1
1425983_x_at	Hipk2	1426426_at	Mak16	1434748_at	Ckap2

1431894_at	Itprip	1429080_at	Mphosph10	1422430_at	Fignl1
1428289_at	Klf9	1451039_at	2610027L16Rik	1438320_s_at	Mcm7
1436387_at	C330006P03Rik	1458097_at	Cobll1	1420028_s_at	Mcm3
1457573_at	Top1	1438606_a_at	Clic4	1416031_s_at	Mcm7
1424457_at	Apbb3	1437785_at	Adamts9	1426652_at	Mcm3
1450722_at	Nup50	1423393_at	Clic4	1448777_at	Mcm2
1438761_a_at	Odc1	1416440_at	Cd164	1434993_at	Fam5c
1438630_x_at	Mat2a	1417040_a_at	Bok	1419452_at	Uchl5
1448802_at	Nufip1	1450928_at	Id4	1417541_at	Hells
1422851_at	Hmga2	1448916_at	Mafg	1429364_at	4930579G24Rik
1450781_at	Hmga2	1448689_at	Rras2	1418727_at	Nup155
1444565_at	BB166591	1456243_x_at	Mcl1	1416737_at	Gys1
1442340_x_at	Cyr61	1428350_at	Ctu2	1448127_at	Rrm1
1438188_x_at	Slc25a29	1420622_a_at	Hspa8	1435292_at	Tbc1d4
1424380_at	Vps37b	1455741_a_at	Ece1	1439269_x_at	Mcm7
1423135_at	Thy1	1423142_a_at	Gtpbp4	1454952_s_at	Ncapd3
1435534_a_at	Tomm20	1415965_at	Scd1	1449705_x_at	Mcm3
1417293_at	Hs6st1	1448158_at	Sdc1	1456280_at	Clspn
1448029_at	Tbx3	1417398_at	Rras2	1439012_a_at	Dck
1451072_a_at	Rnf4	1455398_at	Lrrc8c	1448187_at	Pold1
1451458_at	Tmem2	1434054_at	Mafg	1455654_at	Hjurp
1450780_s_at	Hmga2	1435379_at	Urb2	1417938_at	Rad51ap1
1418033_s_at	Zkscan6	1437177_at	Larp4	1436707_x_at	Ncaph
1425423_at	Glis1	1437279_x_at	Sdc1	1453314_x_at	2610039C10Rik
1456702_x_at	Mat2a	1415943_at	Sdc1	1435737_a_at	Nde1
1418432_at	Cab39	1434304_s_at	Nus1	1438434_at	Arhgap11a
1419209_at	Cxcl1	1460672_at	2410002F23Rik	1448369_at	Pola2
1428457_at	Ttpal	1433656_a_at	Gnl3	1415810_at	Uhrf1
1437626_at	Zfp36l2	1415944_at	Sdc1	1416802_a_at	Cdca5
1437635_at	Dcbld2	1427893_a_at	Pmvk	1424511_at	Aurka
1429897_a_at	D16Ertd472e	1452209_at	Pkp4	1421818_at	Bcl6
1426875_s_at	Srxn1	1424238_at	Sirt7	1437251_at	Cdca2
1422169_a_at	Bdnf	1450742_at	Bysl	1423092_at	Incenp
1423100_at	Fos	1424333_at	Rg9mtd1	1434699_at	G2e3
1416881_at	Mcl1	1420753_at	Tll1	1428483_a_at	2610039C10Rik
1427033_at	Dnmbp	1458126_at	D030041H20Rik	1419513_a_at	Ect2
1433804_at	Jak1	1424051_at	Col4a2	1439510_at	Sgol1
1420024_s_at	Etf1	1434177_at	Ece1	1448191_at	Plk1
1422264_s_at	Klf9	1438802_at	Foxp1	1443161_at	---
1424411_at	Tmem189	1449029_at	Mknk2	1439394_x_at	Cdc20
1448877_at	Dlx2	1423392_at	Clic4	1426349_s_at	Tmpo
1433899_x_at	Tsc22d1	1448413_at	2410016O06Rik	1422628_at	Fam111a
1442606_at	---	1454963_at	Pde12	1452036_a_at	Tmpo
1446196_at	Hmga2	1434200_at	BC010981	1427094_at	Pole2
1419486_at	Foxc1	1452831_s_at	Ppat	1435597_at	Atad5
1419647_a_at	Ier3	1442793_s_at	LOC100046166	1434700_at	G2e3
1419642_at	Purb	1450077_at	Chd1	1441547_at	---
1451680_at	Srxn1	1425921_a_at	1810055G02Rik	1424465_at	Ccdc58
1428254_at	Purb	1456590_x_at	Akr1b3	1424299_at	Oma1
1435483_x_at	Slc25a32	1437223_s_at	Xbp1	1439695_a_at	Kif20b
1418220_at	Foxf2	1419548_at	Kpna1	1422513_at	Ccnf
1428736_at	Gramd3	1421140_a_at	Foxp1	1425734_a_at	Ccdc77
1420888_at	Bcl2l1	1454795_at	Cobll1	1417185_at	Ly6a
1456022_at	Hipk2	1423289_a_at	1810029B16Rik	1416069_at	Pfkp
1423389_at	Smad7	1416835_s_at	Amd1	1418443_at	Xpo1
1432426_a_at	Ube2f	1449670_x_at	Gpr137b	1454694_a_at	Top2a
1429650_at	Stk40	1429596_at	Slc7a6os	1426942_at	Aim1
1423481_at	Riok2	1423566_a_at	Hsph1	1423627_at	Nqo1

1460378_a_at	Tes	1434744_at	Yrdc	1439375_x_at	Aldoa
1442471_at	---	1456566_x_at	Rbm14	1424555_at	9430015G10Rik
1436329_at	Egr3	1425993_a_at	Hsph1	1437109_s_at	Lsm6
1449470_at	Dlx1	1433962_at	Trmt61a	1448627_s_at	Pbk
1455102_at	Larp4	1426286_at	Noc3l	1450899_at	Nedd1
1444064_at	Samhd1	1432059_x_at	5031425E22Rik	1417822_at	D17H6S56E-5
1435128_at	Baiap2	1436979_x_at	Rbm14	1454743_at	Nup205
1421134_at	Areg	1431422_a_at	Dusp14	1419172_at	Dhfr
1418014_a_at	B4galt1	1415964_at	Scd1	1452458_s_at	Ppil5
1416086_at	Tpst2	1450569_a_at	Rbm14	1417118_a_at	Naa10
1423614_at	Lrrc8c	1423904_a_at	Pvr	1423774_a_at	Prc1
1426937_at	6330406I15Rik	1451828_a_at	Acsl4	1424118_a_at	Spc25
1449108_at	Fdx1	1451649_a_at	Wdr75	1416309_at	Nusap1
1460393_a_at	Dusp7	1418226_at	Orc2	1418649_at	Egln3
1427114_at	Ttc19	1455032_at	Ccnyl1	1416757_at	Zwilch
1457823_at	Cyr61	1428694_at	Mir17hg	1435836_at	Pdk1
1439367_x_at	Arf4	1428080_at	Pgam5	1448635_at	Smc2
1419640_at	Purb	1439747_at	Ptges	1435733_x_at	Rnaseh2c
1439837_at	Gigyf2	1448188_at	Ucp2	1452242_at	Cep55
1426691_at	Tjap1	1425348_a_at	Srprb	1429658_a_at	Smc2
1453582_at	Chka	1416998_at	Rrs1	1427707_a_at	Stil
1426567_a_at	Pqlc1	1436541_at	2310008H09Rik	1433893_s_at	Spag5
1458700_at	---	1450871_a_at	Bcat1	1451077_at	LOC100503670
1438386_x_at	Mat2a	1451278_a_at	Mettl11a	1452597_at	2310061C15Rik
1441855_x_at	Cxcl1	1455958_s_at	Pptc7	1416214_at	Mcm4
1442277_at	Chka	1420011_s_at	Xbp1	1436808_x_at	Mcm5
1428535_at	9430020K01Rik	1449505_at	Kpna1	1429660_s_at	Smc2
1455185_s_at	Phf16	1448480_at	Nip7	1415945_at	Mcm5
1427084_a_at	Map4k5	1427997_at	Ndufaf4	1449298_a_at	Pde1a
1457472_at	Gigyf2	1416313_at	Mllt11	1448225_at	Gpaa1
1430491_at	Bop1	1435222_at	Foxp1	1423714_at	Asf1b
1452214_at	Skil	1459741_x_at	Ucp2	1433408_a_at	Mcm10
1455191_x_at	Pip5k1a	1417245_at	Gpr180	1429273_at	Bmper
1437566_at	LOC100505087	1460563_at	Nus1	1416251_at	Mcm6
1416933_at	Por	1436346_at	Cd109	1452917_at	Rfc5
1452009_at	Ttc39b	1438677_at	Pkp4	1452353_at	Gpr155
1436125_at	D16Ertd472e	1416588_at	Ptprn	1437580_s_at	Nek2
1445866_at	Mast4	1428390_at	Wdr43	1452659_at	Dek
1449702_at	Zfand2a	1421142_s_at	Foxp1	1452924_at	Fam83d
1452008_at	Ttc39b	1421141_a_at	Foxp1	1423920_at	Ncaph
1444777_at	Rai14	1449982_at	Il11	1439377_x_at	Cdc20
1435226_at	Rnf19b	1454733_at	Nod1	1417458_s_at	Cks2
1448306_at	Nfkbia	1448782_at	Txndc11	1433862_at	Espl1
1449731_s_at	Nfkbia	1418824_at	Arf6	1430193_at	Casc5
1451163_at	Tinf2	1424019_at	Nop2	1448314_at	Cdk1
1438658_a_at	S1pr3	1428869_at	Nolc1	1422460_at	Mad2l1
1432007_s_at	Ap2a2	1424475_at	Camkk2	1417821_at	D17H6S56E-5
1433891_at	Lgr4	1430111_a_at	Bcat1	1424766_at	Ercc6l
1423694_at	Kctd10	1420886_a_at	Xbp1	1433813_at	Tmem48
1427998_at	Lsm12	1426500_at	Icmt	1448466_at	Cdca5
1429144_at	Gpcpd1	1435953_at	Btaf1	1428519_at	2610528E23Rik
1428636_at	Steap2	1456528_x_at	Ncl	1428480_at	Cdca8
1416693_at	Foxc2	1428844_a_at	Bclaf1	1417910_at	Ccna2
1442671_at	---	1419798_at	2610019E17Rik	1423847_at	Ncapd2
1453851_a_at	Gadd45g	1423040_at	Bzw1	1433725_at	Acvr1b
1428288_at	Klf9	1455195_at	Rps24	1452314_at	Kif11
1417168_a_at	Usp2	1423041_a_at	Bzw1	1424046_at	Bub1
1439122_at	Ddx6	1426987_at	5430417L22Rik	1449699_s_at	C330027C09Rik

1423116_at	Dom3z	1450845_a_at	Bzw1	1424136_a_at	LOC433064
1440678_at	---	1423522_at	Npm3	1448205_at	Ccnb1
1422612_at	Hk2	1454524_at	2310075M01Rik	1423748_at	Pdk1
1440818_s_at	Sf3b1	1433883_at	Tpm4	1428104_at	Tpx2
1424735_at	Slc25a25	1451160_s_at	Pvr	1435938_at	Ckap2l
1449188_at	Midn	1424191_a_at	Tmem41a	1423813_at	Kif22
1434350_at	Csrnp1	1442849_at	Lrp1	1433643_at	Cacna2d1
1424863_a_at	Hipk2	1421346_a_at	Slc6a6	1436723_at	Cenpi
1450710_at	Jarid2	1435280_at	AI452195	1416383_a_at	Pcx
1420088_at	Nfkbia	1458996_at	Itga5	1423775_s_at	Prc1
1421031_a_at	2310016C08Rik	1423401_at	Etv6	1449060_at	Kif2c
1438157_s_at	Nfkbia	1437490_x_at	LOC640502	1442280_at	D2Ertd750e
1448824_at	Ube2j1	1423760_at	Cd44	1452954_at	Ube2c
1424836_a_at	Clasp2	1456516_x_at	LOC640502	1417933_at	Igfbp6
1436584_at	Spry2	1437271_at	Clcf1	1421571_a_at	Ly6c1
1454617_at	Arrdc3	1416756_at	Dnajb1	1449966_s_at	Cab39l
1429114_at	Sestd1	1437270_a_at	Clcf1	AFFX-PyruCa	Pcx
1444279_at	Huwe1	1426871_at	Fbxo33	1415936_at	Bcar3
1439662_at	Homer1	1454703_x_at	Snhg1	1428605_at	Ccdc124
1455574_at	Smg7	1417488_at	Fosl1	1451110_at	Egln1
1437363_at	Homer1	1422243_at	Fgf7	1442454_at	Top2a
1419066_at	Ier5l	1452416_at	Il6ra	1416258_at	Tk1
1457231_at	---	1417602_at	Per2	1433543_at	Anln
1434773_a_at	Slc2a1	1426958_at	Rps9	1449061_a_at	Prim1
1426599_a_at	Slc2a1	1453269_at	Unc5b	1425006_a_at	Vrk1
1417409_at	Jun	1453840_at	Pabpc1	1427161_at	Cenpf
1416686_at	Plod2	1437149_at	Slc6a6	1424278_a_at	Birc5
1416687_at	Plod2	1431037_a_at	Elavl1	1434911_s_at	Arhgap19
1426600_at	Slc2a1	1452483_a_at	Cd44	1453924_a_at	Ptgfr
1447339_at	---	1420990_at	Chd1	1423463_a_at	D2Ertd750e
1458089_at	---	1452118_at	Rrp1b	1419351_a_at	l7Rn6
1441799_at	6030422H21Rik	1422591_at	Tceb3	1451246_s_at	Aurkb
1427188_at	Arih1	1446086_s_at	Gli2	1428900_s_at	Mett5d1
1427189_at	Arih1	1437668_at	Ccrl1	1450842_a_at	Cenpa
1417169_at	Usp2	1418528_a_at	Dad1	1423396_at	Agt
1428306_at	Ddit4	1422844_a_at	Wdr77	1433685_a_at	6430706D22Rik
1418025_at	Bhlhe40	1452091_a_at	Rbm28	1420081_s_at	D2Ertd750e
1436316_at	Klf13	1424924_at	Sec63	1439066_at	Angpt1
1446497_at	---	1426618_a_at	Pomgnt1	1454920_at	Uhrf2
1449138_at	Sf3b1	1454860_x_at	Dad1	1428105_at	Tpx2
1452179_at	Phf17	1440272_at	---	1418184_at	Cenpm
1437638_at	Srrm2	1451026_at	Ftsj3	1453769_at	Ckap2l
1428697_at	Dpp8	1421260_a_at	Srm	1417503_at	Rfc2
1439198_at	---	1418963_at	Fam188a	1450156_a_at	Hmmr
1427932_s_at	1200003I10Rik	1424926_at	Sec63	1420776_a_at	Auh
1419639_at	Efnb2	1428112_at	Manf	1422016_a_at	Cenph
1435137_s_at	1200015M12Rik	1450735_at	Pno1	1417450_a_at	Tacc3
1418571_at	Tnfrsf12a	1423151_at	Dnajb11	1427797_s_at	---
1418572_x_at	Tnfrsf12a	1429191_at	Dhx33	1456077_x_at	Cdc25c
1452418_at	1200016E24Rik	1430138_at	Cd3eap	1438009_at	Gm11276
1420131_s_at	Pttg1ip	1417096_at	Rrp15	1431506_s_at	Ppih
1418148_at	Abhd1	1426932_at	D19Bwg1357e	1417911_at	Ccna2
1453238_s_at	3930401B19Rik	1456597_at	Heatr3	1439040_at	Cenpe
1417625_s_at	Cxcr7	1435656_at	Gmps	1453226_at	Kif18b
1449007_at	Btg3	1416290_a_at	Psmc4	1417506_at	Gmnn
1416531_at	Gsto1	1457983_s_at	Rwdd4a	1429171_a_at	Ncapg
1441519_at	Nid1	1439433_a_at	Slc35a2	1435306_a_at	Kif11
1419641_at	Purb	1418131_at	Samhd1	1452305_s_at	Cenpn

1456036_x_at	Gsto1
1419181_at	Zfp326
1451463_at	Prr5
1423260_at	Id4
1415802_at	Slc16a1
1429204_at	Camk2n2
1440908_at	D030063E12
1451335_at	Plac8
1423259_at	Id4
1439387_x_at	Ctu2
1442322_at	---
1416864_at	Surf6
1437342_x_at	Pttg1ip
1452203_at	Obfc2a
1421854_at	Fgl2
1416359_at	Snx18
1425145_at	Il1rl1
1451466_at	D16Ertd472e
1422317_a_at	Il1rl1
1430623_s_at	Obfc2a
1428219_at	Rybp
1447491_at	---
1419728_at	Cxcl5
1420570_x_at	Tcl1b3
1437711_x_at	Odc1
1454846_at	Utp15
1436637_at	---
1448529_at	Thbd
1455257_at	Itgb3
1455660_at	Csf2rb
1437671_x_at	Prss23
1431057_a_at	Prss23
1421855_at	Fgl2
1417389_at	Gpc1
1416041_at	Sgk1
1418492_at	Grem2
1441030_at	Rai14
1442679_at	---
1451344_at	Tmem119
1419638_at	Efnb2
1421151_a_at	Epha2
1416342_at	Tnc
1453345_at	Nipal1
1458140_at	Slit2
1418075_at	St6galnac4
1420380_at	Ccl2
1448593_at	Wisp1
1421228_at	Ccl7
1448594_at	Wisp1
1459622_at	Gm22
1434458_at	Fst
1421365_at	Fst
1425155_x_at	Csf1
1417394_at	Klf4
1426973_at	Gpr153
1437820_at	Foxs1
1419309_at	Pdpn
1420664_s_at	Procr
1417024_at	Hars
1449221_a_at	Rrbp1
1452037_at	Mgat2
1415828_a_at	Serp1
1424243_at	Rwdd4a
1416106_at	Kti12
1435221_at	Foxp1
1451293_at	Rrp9
1416239_at	Ass1
1460704_at	Rfng
1416852_a_at	Ncdn
1428228_at	Pgm3
1426626_at	Gtf2f2
1423239_at	Impdh1
1439459_x_at	Acly
1416485_at	Timm23
1418872_at	Abcb1b
1415725_at	Rrn3
1423828_at	Fasn
1438922_x_at	Slc25a5
1418079_at	Psme3
1434517_at	Wdfy2
1452171_at	Grwd1
1416423_x_at	Ssb
1439279_at	3110007F17Rik
1426369_at	Far1
1434287_at	Agpat5
1427980_at	4933407C03Rik
1415987_at	Hdlbp
1426371_at	Far1
1426123_a_at	Rrbp1
1450087_a_at	Nolc1
1438962_s_at	Ddx31
1428715_at	Gfpt1
1438360_x_at	Slc25a5
1455841_s_at	Grwd1
1450506_a_at	Aen
1428389_s_at	Wdr43
1428543_at	Ppat
1417511_at	Lyar
1448450_at	Ak2
1428783_at	Prkar2a
1437033_a_at	Skp2
1449211_at	Bpnt1
1448251_at	9030425E11Rik
1421038_a_at	Kcnn4
1455237_at	Usp36
1424001_at	Mki67ip
1424522_at	Heatr1
1434103_at	Slc35e1
1448451_at	Ak2
1451199_at	Qtrtd1
1419163_s_at	Dnajc3
1416234_at	Lrrc59
1437343_x_at	Atad3a
1434239_at	Rrp12
1437478_s_at	Efhd2
1419046_at	Brp16
1438091_a_at	H2afz
1435005_at	Cenpe
1418369_at	Prim1
1418281_at	Rad51
1419467_at	Clec14a
1438214_at	Trps1
1438482_at	Wwp2
1417926_at	Ncapg2
1457445_at	Trps1
1423620_at	Cenpq
1456055_x_at	Pold1
1458374_at	C79407
1416961_at	Bub1b
1453053_at	Cenpw
1416664_at	Cdc20
1438627_x_at	Pgd
1418919_at	Sgol1
1447363_s_at	Bub1b
1437370_at	Sgol2
1419943_s_at	Ccnb1
1448899_s_at	Rad51ap1
1437611_x_at	Kif2c
1451128_s_at	Kif22
1424156_at	Rbl1
1425815_a_at	Hmmr
1417772_at	Grhpr
1422814_at	Aspm
1428518_at	Mlf1ip
1426580_at	Plk4
1423747_a_at	Pdk1
1419838_s_at	Plk4
1428481_s_at	Cdca8
1417457_at	Cks2
1424128_x_at	Aurkb
1449171_at	Ttk
1429150_at	Ccdc77
1424629_at	Brca1
1424971_at	Ccdc99
1437716_x_at	Kif22
1434767_at	C79407
1455730_at	Dlgap5
1416076_at	Ccnb1
1449207_a_at	Kif20a
1455983_at	Cdca2
1416299_at	Shcbp1
1436847_s_at	Cdca8
1429295_s_at	Trip13
1429172_a_at	Ncapg
1448953_at	Blm
1438161_s_at	Rfc4
1419076_a_at	Brca2
1437313_x_at	Hmgb2
1418334_at	Dbf4
1420349_at	Ptgfr
1448650_a_at	Pole
1416155_at	Hmgb3
1426817_at	Mki67
1455990_at	Kif23

1454838_s_at	Pkdcc	1423122_at	Avpi1	1418264_at	Cenpk
1450852_s_at	F2r	1435436_at	Epas1	1428727_at	Cep192
1429310_at	Flrt3	1453727_at	Esf1	1446331_at	Ptgfr
1417395_at	Klf4	1428688_at	Pdcd11	1434286_at	Trps1
1452387_a_at	Amotl2	1426675_at	Tomm70a	1452598_at	Gins1
1443558_s_at	Nt5dc3	1448490_at	Adck4	1449877_s_at	Kifc1
1449335_at	Timp3	1426395_s_at	Eif3j	1416558_at	Melk
1419089_at	Timp3	1456865_x_at	Rrs1	1421963_a_at	Cdc25b
1424268_at	Smox	1437630_at	Lsg1	1452073_at	Fam64a
1453102_at	Flrt3	1448647_at	Man2a1	1417800_at	Parp2
1425154_a_at	Csf1	1419281_a_at	Zfp259	1416162_at	Rad21
1456226_x_at	Ddr1	1423358_at	Ece2	1417445_at	Ndc80
1433842_at	Lrrfip1	1418520_at	Tgoln1	1424292_at	Depdc1a
1429049_at	Nuak2	1455242_at	Foxp1	1433838_at	Dars2
1427057_at	Nt5dc3	1429363_at	Vps37a	1443694_at	Rgs20
1417045_at	Bid	1416874_a_at	Paf1	1418380_at	Terf1
1422705_at	Pmepa1	1423884_at	Cirh1a	1452197_at	Smc4
1422706_at	Pmepa1	1435690_at	2310008H09Rik	1424955_at	Haus1
1449405_at	Tns1	1455330_at	Nol9	1453107_s_at	4933413G19Rik
1424384_a_at	Znrf1	1424244_at	Rwdd4a	1452534_a_at	Hmgb2
1444097_at	Metrnl	1424193_at	Pwp2	1448622_at	Lsm4
1427005_at	Plk2	1450089_a_at	Srprb	1452040_a_at	Cdca3
1442107_at	Flnb	1437359_at	Rnps1	1427276_at	Smc4
1452413_at	C230081A13Rik	1448704_s_at	H47	1419352_at	l7Rn6
1418286_a_at	Efnb1	1423291_s_at	Hyou1	1434828_at	Fam102b
1437700_at	Schip1	1433505_a_at	Lrrc8d	1455033_at	Fam102b
1424356_a_at	Metrnl	1426350_at	Mgat2	1455609_at	Cit
1420979_at	Pak1	1435735_x_at	H47	1438390_s_at	Pttg1
1441679_at	---	1428878_a_at	Pitpnc1	1419705_at	Car5b
1449334_at	Timp3	1452095_a_at	H47	1424105_a_at	Pttg1
1450970_at	Got1	1435066_at	Pitpnc1	1430574_at	Cdkn3
1418424_at	Tnfaip6	1455204_at	Pitpnc1	1450920_at	Ccnb2
1455667_at	Preb	1434418_at	Lass6	1429095_at	Cenpp
1435580_at	C230081A13Rik	1417860_a_at	Spon2	1432361_a_at	Cenpp
1428909_at	A130040M12Rik	1455328_at	Accn2	1431087_at	Spc24
1422771_at	Smad6	1421297_a_at	Cacna1c	1452621_at	Pcbd2
1433605_at	Inpp5a	1455157_a_at	Fam38a	1435575_at	Kntc1
1433184_at	6720477C19Rik	1455287_at	Cdk6	1419153_at	2810417H13Rik
1440999_at	Zfp697	1427347_s_at	Tubb2a	1429156_at	Cenpw
1434033_at	Tle1	1424211_at	Slc25a33	1426767_at	Wdr90
1460411_s_at	Pkdcc	1435338_at	Cdk6	1432591_at	Pappa
1424890_at	Bnc1	1424529_s_at	Cgref1	1448145_at	Wwp2
1448560_at	Bid	1448596_at	Slc6a8	1415849_s_at	Stmn1
1434694_at	Lrrc8a	1450005_x_at	Dlk2	1456097_a_at	Itgb3bp
1435031_at	Tmem120a	1416010_a_at	Ehd1	1448113_at	Stmn1
1451596_a_at	Sphk1	1439030_at	Gmppb	1444071_at	9630013A20Rik
1456888_at	Pfkfb4	1420807_a_at	Dlk2	1450692_at	Kif4
1426625_at	Zfp623	1448175_at	Ehd1	1433604_x_at	Aldoa
1424801_at	Enah	1455943_at	Zfp451	1421546_a_at	Racgap1
1446245_at	---	1429298_at	Ddah1	1427165_at	Il13ra1
1440111_at	---	1416011_x_at	Ehd1	1450081_x_at	Gpi1
1455599_at	Gfod1	1416360_at	Snx18	1451932_a_at	Adamtsl4
1455002_at	Ptp4a1	1460011_at	Cyp26b1	1418442_at	Xpo1
1452083_a_at	Pja1	1455924_at	Rab6b	1424991_s_at	Tyms
1418350_at	Hbegf	1454841_at	Urb1	1439453_x_at	Rnaseh2c
1415803_at	Cx3cl1	1431074_a_at	Pitpnc1	1417427_at	Rnaseh2c
1444340_at	C230066G23Rik	1438317_a_at	Endog	1417971_at	Nrm
1430125_s_at	Pqlc1	1452579_at	Iscu	1451358_a_at	Racgap1

1450850_at	Ezr
1423571_at	S1pr1
1428851_at	1300014I06Rik
1438375_at	Fbln2
1422053_at	Inhba
1426449_a_at	Pja1
1459457_at	Camk2d
1420760_s_at	Ndrg1
1442194_at	AU018552
1437481_at	Trmt61b
1459728_at	Isy1
1446481_at	---
1450214_at	Adora2b
1449857_at	1200011I18Rik
1421811_at	Thbs1
1450931_at	Dock9
1419706_a_at	Akap12
1424923_at	Serpina3g
1460302_at	Thbs1
1442434_at	D8Ertd82e
1433454_at	Abtb2
1444028_s_at	Dock9
1422818_at	Nedd9
1434285_at	Frmd4a
1450932_s_at	Dock9
1457297_at	A430081F14Rik
1442223_at	Enah
1438673_at	Slc4a7
1417612_at	Ier5
1416953_at	Ctgf
1440831_at	Bach1
1438824_at	Slc20a1
1426448_at	Pja1
1457053_at	---
1452445_at	Slc41a2
1450377_at	Thbs1
1441663_at	---
1416250_at	Btg2
1448914_a_at	Csf1
1433453_a_at	Abtb2
1438417_at	Pwwp2b
1419879_s_at	Trim25
1422785_at	Rasa2
1417790_at	Dok1
1417342_at	Ppp1r2
1417434_at	Gpd2
1416156_at	Vcl
1456160_at	---
1438783_at	---
1448568_a_at	Slc20a1
1448684_at	Ppp1r2
1423025_a_at	Iqcj-schip1
1418285_at	Efnb1
1417341_a_at	Ppp1r2
1415797_at	Ddr1
1451591_a_at	Efnb1
1460220_a_at	Csf1
1457492_at	Trio
1420808_at	Gm6768
1459740_s_at	Ucp2
1420501_at	Dnajc1
1452414_s_at	Ccdc86
1416191_at	Sec61a1
1455476_a_at	Gse1
1420646_at	Nus1
1457279_at	LOC100504040
1427464_s_at	Hspa5
1423192_at	Pspc1
1454197_a_at	Ccdc86
1449660_s_at	Coro1c
1455173_at	Gspt1
1442569_at	---
1426471_at	Zfp52
1428273_at	Abhd13
1455400_at	Ddah1
1433506_at	Lrrc8d
1417400_at	Rai14
1419456_at	Dcxr
1415827_a_at	Serp1
1454995_at	Ddah1
1451339_at	Suox
1449870_a_at	Atp6v0a2
1424322_at	Apex2
1433733_a_at	Cry1
1435935_at	2410131K14Rik
1423223_a_at	Prdx6
1422678_at	Dgat2
1452521_a_at	Plaur
1426756_at	Galnt2
1423957_at	Aen
1458847_at	---
1423501_at	Max
1434104_at	Slc35e1
1437500_at	Noc3l
1437859_x_at	Eif5a
1416606_s_at	Nhp2
1416015_s_at	Abce1
1417724_at	Thoc4
1454976_at	Sod2
1415771_at	Ncl
1423756_s_at	Igfbp4
1415800_at	Gja1
1437992_x_at	Gja1
1423398_at	Taf12
1439439_x_at	Eef1d
1416563_at	Ctps
1452915_at	Prkar2a
1416973_at	Nhp2l1
1426946_at	Ipo5
1431893_a_at	Pdss1
1415773_at	Ncl
1452439_s_at	Srsf2
1416147_at	Hspa4
1428788_at	Pgp
1430982_at	Srsf1
1455482_at	Ap2a2
1449191_at	Wfdc12
1445443_at	---
1427105_at	Cenpn
1418152_at	Hmgn5
1422948_s_at	Gm11275
1454909_at	Tacc1
1425668_a_at	St3gal4
1437218_at	Fn1
1426397_at	Tgfbr2
1452540_a_at	Gm11277
1451069_at	Pim3
1430030_at	5330426P16Rik
1418719_at	Haus8
1419397_at	Pola1
1435753_a_at	Nucks1
1455887_at	Alg8
1422470_at	Bnip3
1451263_a_at	Fabp4
1437100_x_at	Pim3
1417023_a_at	Fabp4
1453416_at	Gas2l3
1446951_at	P4ha3
1418072_at	Hist1h2bc
1416613_at	Cyp1b1
1457035_at	AI607873
1417923_at	Pak3
1459902_at	2700007P21Rik
1438988_x_at	Hn1
1456808_at	---
1419006_s_at	Peli2
1425379_at	Hgf

1455862_at	Ubtd2	1452753_at	Foxk2
1418730_at	Rlim	1448178_a_at	Cct3
1445534_at	Flnb	1454640_at	Chchd7
1433768_at	Palld	1460247_a_at	Skp2
1427228_at	Palld	1416024_x_at	Cct3
1448272_at	Btg2	1448192_s_at	Gm5081
1439556_at	Ncam1	1460691_at	Zfp598
1429693_at	Dab2	1456383_at	Rsl1d1
1420498_a_at	Dab2	1428484_at	Osbpl3
1429783_at	Pdlim5	1448707_at	Taf13
1415758_at	Fryl	1433746_at	Wdr3
1436551_at	Fgfr1	1423403_at	Mapkbp1
1450229_at	Med14	1416685_s_at	Fbl
1437061_at	Mbd1	1434543_a_at	Bola2
1421413_a_at	Pdlim5	1423456_at	Bzw2
1457637_at	---	1440051_at	---
1416303_at	Litaf	1415754_at	Polr2f
1452741_s_at	Gpd2	1448469_at	Nid1
1441114_at	9330156P08Rik	1415807_s_at	Srsf2
1429487_at	Ppp1r12a	1433952_at	Tufm
1425811_a_at	Csrp1	1421945_a_at	Rpf2
1435542_s_at	Cttnbp2nl	1422993_s_at	Refbp2
1442919_at	Sh3pxd2b	1448947_at	2810004N23Rik
1437734_at	Ppp1r12a	1435536_at	Ddi2
1444992_at	AI120166	1416605_at	Nhp2
1434181_at	Fermt2	1421491_a_at	Tmem49
1448419_at	Pop4	1433599_at	Baz1a
1447612_x_at	Kdm6b	1448677_at	Cox4nb
1455179_at	Mpp7	1416706_at	Rpe
1418135_at	Aff1	1460667_at	U90926
1438781_at	Tet2	1451833_a_at	Setdb1
1419583_at	Cbx4	1433766_at	Naa25
1435133_at	Ugcg	1433567_at	Gmps
1449773_s_at	Gadd45b	1450401_at	Tgs1
1429639_at	Gpcpd1	1418024_at	Naa15
1425514_at	Pik3r1	1416705_at	Rpe
1438725_at	Med13	1415699_a_at	Gps1
1440443_at	E030016H06Rik	1428163_at	Sar1b
1432216_s_at	Mpp7	1432016_a_at	Idh3a
1435867_at	Jhdm1d	1435372_a_at	Pa2g4
1436498_at	Arih1	1423264_at	Bop1
1434180_at	Fermt2	1417764_at	Ssr1
1456386_at	---	1426674_at	Eif3b
1454158_at	Mpp7	1428870_at	Nolc1
1459917_at	Ggnbp2	1420491_at	Eif2s1
1455166_at	Arl5b	1438510_a_at	Hars
1418892_at	Rhoj	1438178_x_at	Atad3a
1417329_at	Slc23a2	1448604_at	Uck2
1440179_x_at	Rnf217	1418969_at	Skp2
1437741_at	Rab21	1452012_a_at	Exosc1
1450026_a_at	B3gnt2	1423796_at	Sfpq
1437884_at	Arl5b	1416939_at	Ppa1
1418936_at	Maff	1456541_x_at	Atad3a
1454707_at	2310035C23Rik	1438910_a_at	Stom
1450744_at	Ell2	1435333_at	Ndufaf4
1423161_s_at	Spred1	1426290_at	Dimt1
1444651_at	LOC553089	1448230_at	Usp10
1436305_at	Rnf217	1428069_at	Cdca7

1447240_at	---	1417233_at	Chchd4
1436977_at	---	1420867_at	Tmed2
1417357_at	Emd	1416345_at	Timm8a1
1459911_at	Cdr2l	1455261_at	Luc7l
1451041_at	Rock2	1433467_at	Slc7a6
1429251_at	LOC100503505	1451296_x_at	Pabpc4
1458739_at	---	1434545_x_at	Bola2
1423306_at	2010002N04Rik	1431802_a_at	Ept1
1415840_at	Elovl5	1426609_at	Dis3
1437211_x_at	Elovl5	1421528_a_at	Med22
1437396_at	Creb3l2	1426579_at	Gnl2
1422698_s_at	Jarid2	1451753_at	Plxna2
1434080_at	Aebp2	1417235_at	Ehd3
1455876_at	Slc4a7	1438938_x_at	Phb2
1438061_at	4930523C07Rik	1422461_at	Atad3a
1416543_at	Nfe2l2	1416267_at	Scoc
1434966_at	Sfswap	1452829_at	Cad
1436763_a_at	Klf9	1418775_at	AI837181
1440671_at	A130012E19Rik	1437032_x_at	Rbm14
1430043_at	Ttc19	1416020_a_at	Atp5g1
1430407_at	3110035C09Rik	1418222_at	2610024G14Rik
1451612_at	Mt1	1449372_at	Dnajc3
1426415_a_at	Trim25	1426909_at	Uck2
1457268_at	Dot1l	1415750_at	Tbl3
1453283_at	Pgm1	1426496_at	Wdr55
1419655_at	Tle3	1416791_a_at	Nxf1
1416304_at	Litaf	1434438_at	Samhd1
1418970_a_at	Bcl10	1434524_at	Eif2b3
1448587_at	Tbc1d10a	1448144_at	Hnrnpab
1452352_at	Ctla2b	1433887_at	Dnajc3
1428083_at	Neat1	1422497_at	Slc30a5
1420640_at	Jmy	1455174_at	Rps19bp1
1455300_at	Tet2	1424161_at	Ddx27
1424750_at	Zbtb1	1448968_at	Ubfd1
1418971_x_at	Bcl10	1424436_at	Gart
1423160_at	Spred1	1450965_at	Tex261
1418324_at	Fem1b	1416059_at	Sec23b
1419654_at	Tle3	1421012_at	Srprb
1442248_at	---	1434544_at	Bola2
1424644_at	Tbcc	1434235_at	Slc20a2
1445027_at	Cdr2l	1428374_at	Glce
1443027_at	---	1423290_at	Hyou1
1424050_s_at	Fgfr1	1452754_at	Creld2
1424027_at	Pxn	1416362_a_at	Fkbp4
1427199_at	Fryl	1452878_at	Prkce
1425911_a_at	Fgfr1	1428315_at	Ebna1bp2
1436801_x_at	Cdc42ep4	1426676_s_at	Tomm70a
1418323_at	Fem1b	1448132_at	Slc19a1
1456135_s_at	Pxn	1460433_at	Entpd6
1437900_at	4930523C07Rik	1417873_at	Pwp1
1418295_s_at	Dgat1	1420138_at	Slc19a1
1431299_a_at	2310014H01Rik	1437471_at	Lrrc45
1433494_at	Dos	1459987_s_at	Cct3
1452837_at	Lpin2	1416068_at	Kars
1438370_x_at	Dos	1417192_at	Tomm70a
1449311_at	Bach1	1451262_a_at	Aimp2
1425576_at	Ahcyl1	1426931_s_at	D19Bwg1357e
1418145_at	Tfip11	1456396_at	---

1455616_at	Aebp2	1434173_s_at	D19Bwg1357e
1456344_at	Tnc	1430999_a_at	Scoc
1457528_at	Slc4a7	1449645_s_at	Cct3
1433634_at	Irf2bp2	1416890_at	Wdr74
1442359_at	Steap2	1441788_s_at	Dkc1
1456150_at	Jhdm1d	1456523_at	Gm2115
1423622_a_at	Ccnl1	1420497_a_at	Cebpz
1425640_at	Aff1	1416684_at	Fbl
1457167_at	Med14	1417053_at	Phb
1432478_a_at	Rnf19b	1438853_x_at	Ddx54
1422931_at	Fosl2	1415710_at	Cox18
1416432_at	Pfkfb3	1454814_s_at	Gm5081
1418256_at	Srf	1416052_at	Prps1
1446167_at	---	1453257_at	Agpat5
1427359_at	Jhdm1d	1416346_at	Timm8a1
1437173_at	S1pr3	1451170_s_at	Nomo1
1431162_a_at	Enah	1439740_s_at	Uck2
1433542_at	Inpp5f	1417057_a_at	Lamp3
1431256_at	5033423O07Rik	1417725_a_at	Sssca1
1419247_at	Rgs2	1460730_at	Eif2b1
1419248_at	Rgs2	1417692_at	Drg2
1447830_s_at	Rgs2	1434710_at	Dhx29
1424748_at	Galnt11	1438016_at	Dkc1
1428164_at	Nudt9	1415916_a_at	Mthfd1
1459253_at	1700023H06Rik	1438168_x_at	Ddx39
1457276_at	Sik2	1419097_a_at	Stom
1448694_at	Jun	1416931_at	Nif3l1
1432686_at	4833406M21Rik	1432211_a_at	Fbxo9
1419874_x_at	Zbtb16	1416421_a_at	Ssb
1436338_at	---	1435057_x_at	Polr1e
1418678_at	Has2	1424323_at	Noc2l
1427061_at	Rbbp8	1448315_a_at	Pycr2
1422946_a_at	Dnmt1	1451169_at	Nomo1
1449169_at	Has2	1436310_at	Gemin5
1424212_at	9430023L20Rik	1424151_at	Aimp2
1430362_at	5730409N24Rik	1438015_at	Dkc1
1425255_s_at	Hnrpll	1417657_s_at	Dnajc2
1439163_at	Zbtb16	1416792_at	Ppm1g
1442025_a_at	---	1422455_s_at	Nsf
1435122_x_at	Dnmt1	1434681_at	Txlng
1459289_at	---	1450400_at	Tgs1
1424613_at	Gprc5b	1423060_at	Pa2g4
1420965_a_at	Enc1	1435244_at	Vav2
1417947_at	Pcna	1448907_at	Thop1
1450061_at	Enc1	1450724_at	Fam126a
1457404_at	Nfkbiz	1428106_at	1300001I01Rik
1418133_at	Bcl3	1448126_at	Fam60a
1434603_at	Med13l	1424620_at	Nop16
1434602_at	Med13l	1426118_a_at	Tomm40
1435460_at	Prkg2	1448794_s_at	Dnajc2
1451411_at	Gprc5b	1417807_at	Ufsp1
1427035_at	Slc39a14	1416038_at	Snd1
1448728_a_at	Nfkbiz	1434243_s_at	Tomm70a
1429758_at	1700017B05Rik	1452830_s_at	Cad
1438660_at	Gcnt2	1419058_at	Polr1e
1429399_at	Rnf125	1435985_at	Farp2
1458989_at	---	1455060_at	G3bp1
1435589_at	Ccdc85b	1426631_at	Pus7

1435084_at	C730049O14Rik	1425820_x_at	Gpatch4
1442700_at	Pde4b	1454817_at	Utp18
1447877_x_at	Dnmt1	1429092_at	Vkorc1l1
1447063 at	1700017B05Rik	1435076 at	Fam57a
		1424533_a_at	Ccdc137
		1452429_s_at	Abcf1
		1448582_at	Ctnnbl1
		1449001_at	Ivd
		1455787 x at	Minpp1
		1454142_a_at	Pwp1
		1424659_at	Slit2
		1415733_a_at	Tomm5
		1421160_a_at	Rfng
		1425561 at	Trnt1
		1418057_at	Tiam1
		1449886_a_at	Timm9
		1452048_at	Mrpl12
		1416750_at	Sigmar1
		1416448 at	Itpa
		1438650_x_at	Gja1
		1454904_at	Mtm1
		1451166_a_at	Ccdc101
		1434537_at	Slco3a1
		1423643 at	Ddx39
		1423334_at	Ergic1
		1429456_a_at	Polr3e
		1423333_at	Ergic1
		1455832_a_at	Umps
		1417775 at	Polr1a
		1416400_at	Pycrl
		1433663_s_at	Ncbp1
		1417236_at	Ehd3
		1447903_x_at	Ap1s2
		1456653 a at	Mthfd1l
		1443794_x_at	Noc4l
		1416014_at	Abce1
		1452657_at	Ap1s2
		1441327 a at	Ssr1
		1417193_at	Sod2
		1434222_at	Sipa1l1
		1427305_at	Piga
		1418509_at	Cbr2
		1452919 a at	Pgp
		1448610_a_at	Sod2
		1424459_at	Lpcat1
		1425497_a_at	Prpf4b
		1423241_a_at	Tfdp1
		1416962 at	Rcc1
		1438095_x_at	Noc4l
		1423371_at	Pole4
		1416730_at	Rcl1
		1423709_s_at	Farsb
		1419680 a at	Elac2
		1417890_at	Pdxp
		1455655_a_at	Tardbp
		1448633_at	Prpf31
		1450854_at	Pa2g4
		1422482 at	Ruvbl2

1424140_at	Gale
1440299_at	E330016A19Rik
1420142_s_at	Pa2g4
1457281_at	Dnajc21
1428506_at	Atic
1423633_at	Snrnp48
1418030_at	Slco3a1
1450848_at	Dap3
1449140_at	Nudcd2
1428476_a_at	Elac2
1416445_at	Fam98a
1451347_at	Ino80e
1422753_a_at	Polr3k
1428477_at	Elac2
1426513_at	Rbm28
1422796_at	Prep
1415917_at	Mthfd1
1416283_at	Gart
1425830_a_at	Cinp
1448918_at	Slco3a1
1439266_a_at	Polr3k
1423723_s_at	Tardbp
1415772_at	Ncl
1451956_a_at	Sigmar1
1433748_at	Zdhhc18
1440799_s_at	Farp2
1424147_at	Ahsa1
1438376_s_at	Trim27
1437520_a_at	Nup85
1437828_s_at	Wdr46
1422456_at	Nsf
1424907_a_at	Farsa
1416376_at	Tmem97
1423430_at	Mybbp1a
1449040_a_at	Sephs2
1456375_x_at	Trim27
1426607_at	Gm7120
1424203_at	Ncln
1424314_at	Prpf3
1417581_at	Dhodh
1434221_at	Mical3
1460215_at	Polr1a
1422489_at	Mogs
1426727_s_at	Gm8801
1460241_a_at	St3gal5
1428800_a_at	Pus7l
1423740_a_at	Rbm10
1442590_at	Tnfrsf22
1426726_at	Gm8801
1434859_at	Umps
1457083_at	---
1437067_at	Phtf2
1426933_at	Oxsr1
1439516_at	2610201A13Rik
1422709_a_at	Wdr46
1416915_at	Msh6
1416492_at	Ccne1
1460444_at	Arrb1

1419359_at	Hexim1
1425498_at	Prpf4b
1417582_s_at	Dhodh
1452811_at	Atic
1417191_at	Dnajb9
1439407_x_at	Tagln2
1417420_at	Ccnd1
1448698_at	Ccnd1
1417419_at	Ccnd1
1435928_at	6430548M08Rik
1425896_a_at	Fbn1
1434830_at	Mxd1
1419550_a_at	Stk39
1460208_at	Fbn1
1426074_at	---
1437274_at	Copa
1429772_at	Plxna2
1434512_x_at	Srsf3
1434791_at	Atp6v0a2
1430515_s_at	Aasdhppt
1432164_a_at	Gcsh
1434135_at	B3galnt2
1433751_at	Slc39a10
1452768_at	Tex261
1443870_at	Abcc4
1448691_at	Ubqln4
1416126_at	Polr1b
1437373_at	Aen
1448769_at	Slc35b1
1426940_at	Sidt2
1454842_a_at	B3galnt2
1433496_at	Glt25d1
1459842_x_at	Nubp2
1440303_at	Slc7a6os
1428820_at	Mapre1
1448563_at	Phb
1434552_at	Wdr77
1418206_at	Sdf2l1
1448646_at	Wdr12
1434940_x_at	Rgs19
1454659_at	Dctd
1417212_at	Fam195a
1423621_a_at	Slc33a1
1452872_at	Ank3

Echinomycin inhibits adipogenesis in 3T3-L1 cells in a HIF-independent manner
Junna Yamaguchi, Tetsuhiro Tanaka, Hisako Saito, Seitaro Nomura, Hiroyuki Aburatani, Hironori Waki, Takashi Kadowaki, Masaomi Nangaku

Table S2. The list of 111 probes in the microarray analysis (upregulated with MDI and suppressed with echinomycin both at 2 and 6 hr)

Probe Set ID	Gene Symbol
1450714_at	Azin1
1428393_at	Nrn1
1427844_a_at	Cebpb
1437247_at	Fosl2
1417483_at	Nfkbiz
1424938_at	Steap1
1451532_s_at	Steap1
1449851_at	Per1
1416881_at	Mcl1
1456702_x_at	Mat2a
1429758_at	1700017B05Rik
1455870_at	Akap2
1437742_at	Rab21
1418220_at	Foxf2
1420888_at	Bcl2l1
1426825_at	Fmnl3
1422264_s_at	Klf9
1429399_at	Rnf125
1417902_at	Slc19a2
1449169_at	Has2
1422612_at	Hk2
1435483_x_at	Slc25a32
1418678_at	Has2
1427931_s_at	Pdxk
1427930_at	Pdxk
1417487_at	Fosl1
1422851_at	Hmga2
1442213_at	LOC552908
1439168_at	Camk2d
1450780_s_at	Hmga2
1416743_at	LOC640502 /// Uap1
1416125_at	Fkbp5
1450781_at	Hmga2
1418946_at	St3gal1
1422751_at	Tle1
1437490_x_at	LOC640502 /// Uap1
1456516_x_at	LOC640502 /// Uap1
1416745_x_at	LOC640502
1423401_at	Etv6
1448689_at	Rras2
1416744_at	LOC640502
1433531_at	Acsl4
1434602_at	Med13l
1430623_s_at	Obfc2a
1435280_at	AI452195
1426337_a_at	Tead4
1418175_at	Vdr
1437635_at	Dcbld2
1457404_at	Nfkbiz
1434967_at	Zswim6
1455660_at	Csf2rb

1434261_at	Sipa1l2
1447063_at	1700017B05Rik
1419766_at	Sik1
1444064_at	Samhd1
1430362_at	5730409N24Rik
1423267_s_at	Itga5
1459722_at	Zswim6
1424296_at	Gclc
1418911_s_at	Acsl4
1434384_at	Nrip1
1425423_at	Glis1
1418879_at	Fam110c
1418176_at	Vdr
1422771_at	Smad6
1439163_at	Zbtb16
1441030_at	Rai14
1455959_s_at	Gclc
1448231_at	Fkbp5
1420150_at	Spsb1
1424613_at	Gprc5b
1449089_at	Nrip1
1417621_at	Nfatc1
1441823_at	Zmiz1
1451739_at	Klf5
1418469_at	Nrip1
1418901_at	Cebpb
1448728_a_at	Nfkbiz
1451021_a_at	Klf5
1439797_at	Ppard
1448029_at	Tbx3
1430352_at	Adamts9
1437626_at	Zfp36l2
1427114_at	Ttc19
1457276_at	Sik2
1451819_at	Zswim6
1451177_at	Dnajb4
1431734_a_at	Dnajb4
1435084_at	C730049O14Rik
1439279_at	3110007F17Rik
1455904_at	Gas5 /// Snord47
1456381_x_at	Mcl1
1416880_at	Mcl1
1449007_at	Btg3 /// Gm7334
1437527_x_at	Mcl1
1456243_x_at	Mcl1
1431182_at	Hspa8 /// LOC624853
1439837_at	Gigyf2
1426958_at	Rps9
1428694_at	Mir17hg
1448503_at	Mcl1
1418133_at	Bcl3
1416067_at	Ifrd1
1451982_at	Map2k4
1422243_at	Fgf7
1453906_at	Med13l
1426233_at	Map2k4
1438405_at	Fgf7
1437785_at	Adamts9

1439030_at	Gmppb
1449268_at	Gfpt1

Supplementary Figure (Uncropped images for the blots)

Figure 2C. first row original figure

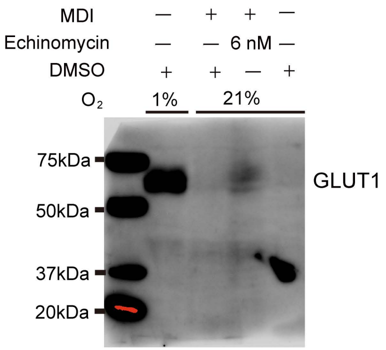

Figure 2C. second row original figure

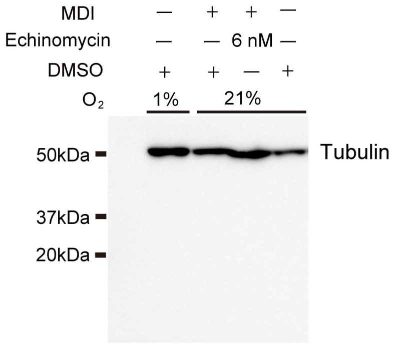

Figure 2D (left). original figure

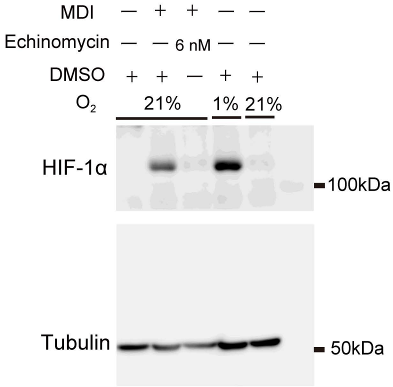

Figure 2D (right). original figure

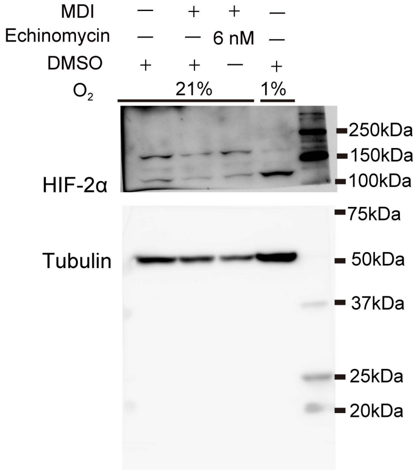

Figure 5A. first row original figure

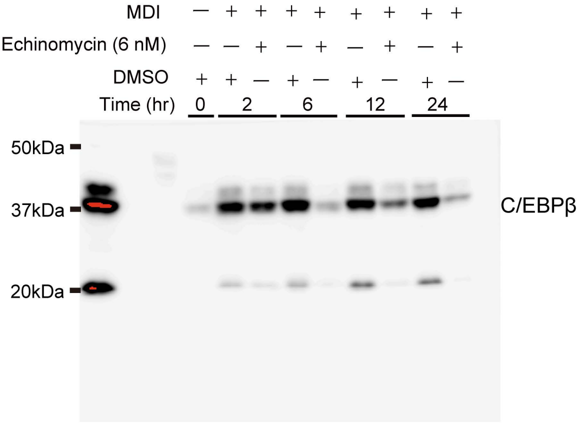

Figure 5A. second row original figure

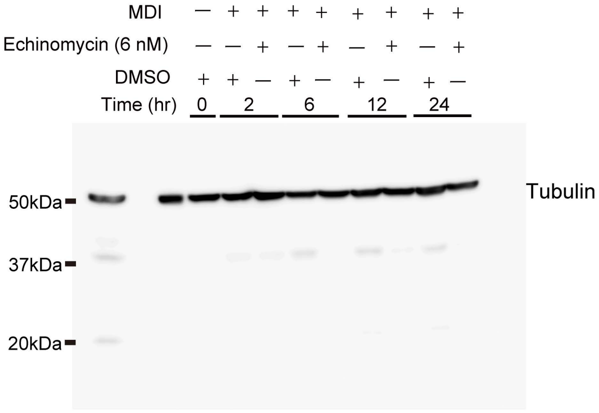

Figure 5C (right). first row original figure

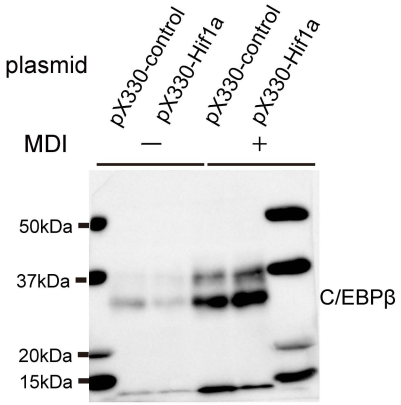

Figure 5C (right). second row original figure

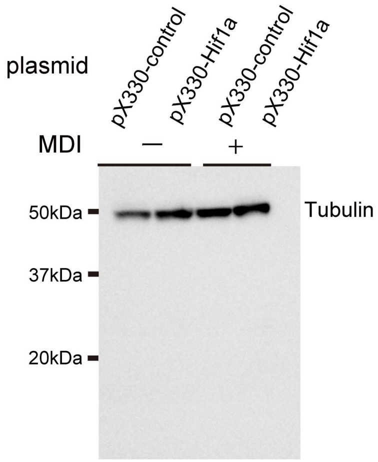

Figure 5D (left). first row original figure

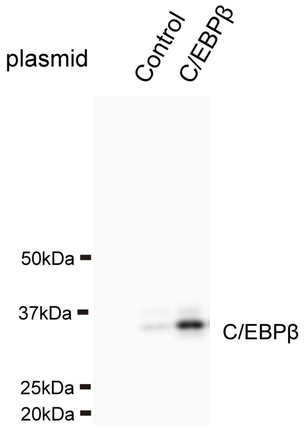

Figure 5D (left). second row original figure

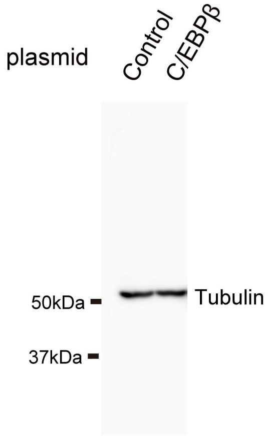

Supplementary Figure (continued) Uncropped images for the blots.
